# Phenotypic characterisation, quality of life and prevalence of idiopathic epilepsy and epilepsy of unknown cause in the nine Dutch dog breeds

**DOI:** 10.3389/fvets.2026.1897489

**Published:** 2026-08-27

**Authors:** Tippy Rolink, Anne van Rooijen, Montserrat Diaz Espineira, Bart Broeckx, Sofie F. M. Bhatti, Paul J. J. Mandigers

**Affiliations:** 1 Department of Clinical Sciences, Faculty of Veterinary Medicine, Utrecht University, Utrecht, Netherlands; 2 Laboratory of Animal Genetics, Department of Veterinary and Biosciences, Faculty of Veterinary Medicine, Ghent University, Ghent, Belgium; 3 Center of Clinical Genetics for Companion Animals, Faculty of Veterinary Medicine, Ghent University, Ghent, Belgium; 4 Small Animal Department, Faculty of Veterinary Medicine, Ghent University, Ghent, Belgium; 5 Evidensia Referral Hospital, Arnhem, Netherlands

**Keywords:** Dutch Partridge dog, Dutch Shepherd, Dutch Smoushound, Kooiker dog, Markiesje dog, Saarloos Wolfdog, Schapendoes dog, Stabyhound Wetterhoun

## Abstract

**Introduction:**

Canine epilepsy is a common and complex neurological disease that occurs in almost all dogs. In the Netherlands, there are nine pedigree dog breeds, and all of them have numerous small populations, with moderate to high inbreeding coefficients. The goal of this study is to characterise epilepsy within these breeds and to estimate the prevalence for each of these nine breeds: Dutch Partridge dog, three variants of the Dutch Shepherd, Dutch Smoushound, Kooiker dog, Markiesje dog, Saarloos Wolfdog, Schapendoes dog, Stabyhoun, and Wetterhoun.

**Method:**

An online questionnaire was distributed to establish the phenotypic characterisation of idiopathic epilepsy (IE) and epilepsy of unknown cause (EUC). The prevalence was estimated using data from breeders’ associations, as well as cases seen by the investigators but unknown to the breed clubs.

**Results:**

The estimated prevalence of IE and EUC was for the Dutch Partridge dog 2.63%, short haired Dutch Shepherd 0.72%, rough haired Dutch Shepherd 1.21%, long haired Dutch Shepherd 0.42%, the Dutch Smoushound 2.22%, Kooiker dog 0.4%, Markiesje dog 0.84%, Saarloos Wolfdog 1.9%, Schapendoes dog 0.23%, Stabyhoun 1.5%, and Wetterhoun 2.8%. All breed clubs use breeding advice to reduce the frequency of IE/EUC, and within the Dutch Shepherd shorthaired, Kooiker dog, Smoushound, and Saarloos Wolfdog, the frequency has dropped over the years. Within all breeds, generalised tonic–clonic seizures (GTCS) were the most frequently observed type of seizures next to cluster seizures (CS) and status epilepticus (SE). Roughly, pre-ictal and post-ictal signs could be observed in half of all epileptic dogs. Anti-seizure medication (ASM) was administered to 78% of the dogs, and 70% of those experienced side effects. Only in the Wetterhoun was the need for an anti-seizure medication (ASM) less. Less than 50% of the Wetterhouns needed an ASM. The most common ASM across all breeds was phenobarbital (PB).

**Conclusion:**

Prevalence varied across the nine breeds, regardless of their population size. The phenotypic characteristics of IE/EUC are comparable with those of other dog breeds. Breeding strategy appeared to be the most effective in the Dutch Smoushound and Kooiker dog.

## Introduction

Canine epilepsy is a common and complex neurological disease that occurs in almost all dogs (1, 2). The International Veterinary Epilepsy Task Force (IVETF) published a classification of epilepsy, distinguishing idiopathic, structural and reactive forms (3). Idiopathic epilepsy (IE) is further subdivided into genetic, suspected genetic or epilepsy of unknown cause (EUC). The term genetic epilepsy is used when a genetic aetiological association has been identified, whereas suspected genetic epilepsy indicates a high degree of suspicion for a genetic background, but no such association has been found (3, 4). Dogs classified as suffering from EUC are either younger than 6 months or older than 6 years, with no underlying cause identified (3, 4). The epilepsy observed in these dogs may still be of genetic origin, as described in the Irish Setter and Labradoodle (5, 6). In both structural epilepsy and reactive seizures, a cause has been established (4).

The prevalence of IE in the general dog population is estimated at 0.60–0.75% (2, 7). However, prevalence varies by country, as some affected breeds, such as the Border Collie, are overrepresented in certain countries, and IE is most likely more prevalent in the Border Collie than in other breeds (1, 8). If, within a breed, IE occurs at a higher frequency than the general estimate, breed clubs generally take preventive measures. For this reason, it is very important that all cases of IE/EUC are reported to them. The actions a breed club may take vary by club and breed. For instance, the Dutch Border Collie Club excludes the affected dog and its parents and aims to reduce the risk of affected offspring. The Dutch Australian Breed Club adds to this rule that dogs used for breeding must be older than 3 years. As most dogs suffering from epilepsy show their first seizure before the age of three, they therefore reduce the risk of accidentally using a dog that turns out to be an epilepsy case at a later age. The Dutch Kooiker dog club calculates the risk of accidentally crossing dogs related to known epilepsy cases (9). For instance, if a sibling of an epilepsy case is used, the risk of this dog being genetically affected is probably high compared with a dog with no known relation to an epilepsy case. They have used this method successfully for hereditary necrotising myelopathy and have reduced the incidence from 2.4 to 0.4% over approximately 25 years (10, 11). With the discovery of the causative mutation of this disease, it is now fully eradicated from this population (11). But without a genetic test or marker, breeds with a high frequency of affected dogs face real challenges in reducing the frequency of affected dogs (12). However, despite these challenges, it is unethical not to take preventive action, and therefore, breed clubs are motivated to set rules for their breeding stock (9, 13).

In the Netherlands, there are nine pedigree dog breeds, of which eight are FCI-registered (14), and all of them have numerous small populations (see Table 1), with moderate to high inbreeding coefficients (15–19). The breeds include a typical hunting dog, the Dutch Partridge dog; a retriever, the Wetterhoun; four Dutch Shepherds, a short-haired, long-haired and rough-haired variety; and the Schapendoes dog. Two breeds had specific duties, such as the Dutch Smoushound, used as a small hunting dog, and the Kooiker dog, used in duck decoys. The Markiesje dog is the only companion breed. However, all of these breeds date from centuries ago, except for one, the Saarloos Wolfdog, created in the early 1932s by Leendert Saarloos, who used a wolf and a German Shepherd to create a new breed (14). The Kooiker dog was nearly extinct and was re-established after the Second World War by retrieving dogs resembling the Kooiker dog (10, 16). Similar actions were taken for the Dutch Smoushound and Markiesje dog in the late 70s and 80s of the previous century (14). Nowadays, most of these breeds can be found in several European countries and occasionally in countries outside Europe, but their numbers remain very low. The breeds with the fewest dogs are the Saarloos Wolfdog and Wetterhoun. The breed with the largest number of dogs alive is most likely the Kooiker dog, with estimates of 17.000 dogs alive (19). All of these breeds have been affected by several heritable diseases, including epilepsy, polymyositis, cardiac diseases, von Willebrand factor deficiency, and others (2, 10, 13, 15, 20–24), that have all diminished the breeding stock.

**Table 1:** Prevalence of IE/EUC for each breed.

IE/EUC cases per time period	Partridge dog	Dutch Shepherd			Kooiker dog	Markiesje	Saarloos Wolfdog	Schapendoes	Smous-hound	Stabyhoun	Wetterhoun
		Short haired	Rough haired	Long haired							
Time period	1970–2023 53 yrs	1991–2020 29 yrs	2015–2023 8 yrs	2010–2023 13 yrs	1980–2020 41 yrs	2006–2023 17 yrs	1980–2021 41 yrs	2015–2023 8 yrs	2004–2023 19 yrs	1978–2021 43 yrs	1979–2020 42 yrs
IE/EUC cases	660	31	3	2	128	18	43	19	50	215	109
Total registered dogs	25,135	4,455	249	477	31,788	2,132	2,257	8,232	2,252	14,192	3,841
Percentage	2.6%	0.7%	1.2%	0.4%	0.4%	0.8	1.9%	0.2%	2.2%	1.5%	2.8%

Managing a small breeding stock means that a breed club has to set priorities. Diseases with very low prevalence are clearly less important than those with high prevalence, provided their impact on animal welfare is low. For this reason, in 2017 the last author of this manuscript conducted a study investigating the frequency of IE across the nine Dutch breeds (13). A breed with a high prevalence of IE appeared to be the Dutch Partridge dog, and for this reason, a genetic study investigating IE was performed. A genome-wide association study revealed a possible risk locus on chromosome 12. However, sequencing the GRIK2 candidate gene revealed no variants of interest. A variant in CCDC85A on chromosome 10 was discovered, and dogs homozygous for the variant (T/T) had an increased risk of developing IE (OR: 6.0; 95% CI: 1.6–22.6). This variant was classified as likely pathogenic according to American College of Medical Genetics and Genomics (ACMG) guidelines, but the authors indicated that further research is necessary before the risk locus or the CCDC85A variant can be used for breeding decisions (24).

However, neither this publication nor the earlier publication by Mandigers (13) described the phenotypic characterisation of IE in the Dutch breeds. Knowledge of phenotypic characterisation is important because it informs diagnostic approaches and treatment, and is necessary for (epi-)genetic studies. For instance, Border Collies and Great Swiss Mountain dogs with IE respond less to treatment, have a higher frequency of seizures, and are more likely to develop cluster seizures (CS) and/or status epilepticus (SE) (1, 8, 25, 26). Understanding the phenotypic characterisation of a breed not only helps to improve treatment and diagnosis but also prepares owners for what to expect. The goal of this study is to characterise epilepsy in these breeds, estimate the prevalence of epilepsy in each of the nine breeds, and investigate the disease’s evolution over time.

## Materials and methods

### Dogs

All included dogs were purebred or descended from two purebred parents of one of the nine Dutch breeds. All dogs were privately owned, and participating owners provided informed consent to participate in the study. Approval from the ethics committee of the animal welfare body was requested but was not required, as all dogs included were referred for clinical reasons. To retrieve the dogs, three options were possible. The first group of dogs included dogs that were either seen earlier by one of the authors at the Department of Clinical Sciences, Faculty of Veterinary Medicine, Utrecht University, The Netherlands; the Small Animal Department, Faculty of Veterinary Medicine, Ghent University, Merelbeke, Belgium; or Evidensia Small Animal Hospital Arnhem, The Netherlands. All of these dogs were registered, with informed consent, in the central database of the Expertise Centre of Genetics at the Faculty of Veterinary Medicine, Utrecht University, The Netherlands. The second group was obtained by direct mailing from the breed clubs. The clubs kept records of reported epilepsy cases, and owners were contacted with the club’s help. Again, all of these owners gave informed consent prior to participating. The third group comprised unreported cases identified by the breeders’ associations through advertisements on various social media platforms, forums, and their websites, inviting owners to participate in this study. Again, all of these owners gave informed consent prior to participating.

### Inclusion criteria

Only dogs with at least 2 epileptic seizures were included. The classification and diagnostic criteria described by the IVETF were applied (3, 4). Hence, a dog could only be included if there was a suitable semiology, history, and unremarkable physical and neurological examination, without an indication for reactive seizures or structural epilepsy. Diagnostic level of confidence was classified as IE TIER I if the age of onset (AoO) was between 6 months and 6 years of age and additional blood-, and urine tests revealed no abnormalities, TIER II if MRI and cerebrospinal fluid analysis were without pathological changes (3, 4). A dog was classified, in this study, as EUC if the dog was either younger than 6 months or older than 6 years at the time of seizure onset, and reactive as well as structural causes were excluded (TIER II level of confidence) (3). If the semiology, history and physical examination were without pathological significance, the dog had been suffering from seizures over a period of at least 1 year without indication for a reactive or structural epilepsy, but the TIER I diagnostic criteria were not met (for instance, a blood examination or urine analysis was missing), the dog was classified as IE TIER 0.

### Exclusion criteria

All responses that lacked essential information were excluded. The following information was considered essential: breed, sex and neutering status, whether the dog was alive or deceased, cause, seizure type, and total number of seizures. Furthermore, all dogs with an abnormal physical and/or neurological examination, abnormal MRI findings, and/or a history suggesting it was not an IE or EUC were excluded.

### Phenotypic characterisation

All owners were asked to complete a web-based questionnaire created with Qualtrics® survey software^1^, which was used in earlier studies by our group (1, 6, 8). The questionnaire, in Dutch, consisted of over 100 questions and was divided into nine sections. Some questions were mandatory to complete, while others could be skipped if they were considered less essential to the study. For instance, questions on type of food and vaccination brand. The first section focused on general information about the dog: name, breed, sex and neuter status, date of birth, pedigree and registration, alive/dead, age and reason for death (if applicable), kinship to other dogs with epilepsy, character, vaccination status and use of anti-parasitic drugs. The second section contained questions about the date of the first seizure, the number of seizures, the type of seizures (generalised tonic–clonic seizures (GTCS), focal seizures (FS) or paroxysmal dyskinesia (PD)) and the cause (idiopathic, reactive or structural). The third section covered additional information on the seizures: questions about possible triggers, the time treatment started, the occurrence and duration of the pre-ictal phase, the type of pre-ictal signs, the predictability and circumstances of seizures, consciousness during the seizure, contact with the dog during the seizure, and the average, longest, and shortest seizure duration. The fourth section focused on the semiology of the seizures: questions about the type of ictal signs, the uniformity of seizures, behaviour between seizures, any left- or right-sided differences, and the ability to shorten the seizure. The fifth section was on the post-ictal phase: questions about recognition of the phase, whether the dog remembers the seizure, duration, responsiveness during the post-ictal phase, and the type of signs. The sixth section addressed the veterinarian’s role: questions about diagnostic methods and other problems affecting the dog. The seventh section covered frequency and severity: questions about the number of seizures, the occurrence and number of CS and SE, and the severity of GTCS and FS. The eighth section focused on medication: questions about the type of anti-seizure medication (ASM), emergency medication, side-effects, diet and diet change, and alternative treatment. The last section used a scale to score the severity of seizures and side-effects (0 = not severe, 10 = severe), fear of leaving the dog at home (0 = no problem, 10 = dog cannot be left alone), owner Quality of Life (QoL; 0 = no QoL, 10 = optimal QoL), whether care is worth it (0 = big problem, 10 = no problem), dog QoL (0 = no QoL, 10 = optimal QoL), and which phase is the worst. The questions were either yes/no, multiple-choice, rating-scale, or open-ended. Some questions were shown only depending on the previously given answers, and some questions were compulsory. Medical terms such as GTCS, FS and PD were explained using YouTube videos. An example of the original questionnaire in Dutch and the previously used translated questionnaire can be found in Appendices 1 and 2. The data was automatically transferred to Microsoft® Excel 2024. The Excel file was checked for duplicates, and if any were present, only the most complete entry was kept.

### Prevalence

To estimate the prevalence, the total number of purebred dogs registered by the breed club as suffering from IE/EUC over a specific timeframe was first calculated. Second, the list was completed by adding purebred dogs suffering from IE/EUC that were not known to the breed club because the owners refused to reveal the dogs’ identities to the breeders’ association. This number was compared with the total number of registered purebred dogs of that breed during the same timeframe.

### Data analysis

The data obtained from the questionnaire and patient records used for the phenotypic characterisation were recorded in a Microsoft® Excel database. The reliability of responses was determined manually and checked for compliance with the inclusion and exclusion criteria. Data was analysed using RStudio® (27). First, a descriptive analysis was conducted. Subsequently, the data were analysed for statistical correlation. For gender distribution, significance was tested with a one-sample binomial test. Significance between two nominal variables was tested with a chi-square test (if values were ≥5) or a Fisher’s exact test. Numerical variables were first tested for normal distribution. After this, a student’s t-Test (for normally distributed data) or a Mann–Whitney U-test (for non-normally distributed data) was used. Results were considered statistically significant if *p* < 0.05. Data were presented as mean ± standard deviation as well as median and range. Not all questions were mandatory to be filled in. In the results section, the total number of included dogs is reported first, but if for a specific item only a smaller number was retrieved, this is indicated by adding the number of owners or dogs that provided this information (Table 2).

**Table 2:** Phenotypic characteristics of IE/EUC for each breed.

Phenotypic characterisation	Partridge dog	Dutch Shepherd	Kooiker dog	Markiesje	Saarloos Wolfdog	Schapendoes	Smous-hound	Stabyhoun	Wetterhoun
Number of dogs	57	5	14	2	10	4	4	13	9
♂/ ♀ ratio	33 vs. 24	3 vs. 2	8 vs. 6	1 vs. 1	7 vs. 3	4 vs. 0	2 vs. 2	8 vs. 5	5 vs. 4
AoO in mths	44 ± 23	46 ± 22	38 ± 20	27 34	41 ± 22	59 ± 18	24 ± 33	39 ± 18	30 ± 18
Triggers present	38%	possible	42%	50%	possible	possible	50%	possible	possible
Ictus duration (minutes)	5.5 ± 3.6	2.5 ± 0.7	2.0 ± 1.2	5	2.9 ± 3.0	3.0 ± 1.7	6.7 ± 7.4	2.9 ± 2.1	3.7 ± 1.2
Frequency / year	6.8 ± 11.3	3 ± 3.4	33 ± 47	9.5	21 ± 19	2.1 ± 2.0	1.1 ± 0.6	6.3 ± 8.3	2.2 ± 1.9
CS present	49%	40%	83%		60%	yes	yes	61.5%	22%
SE present	21%	40%	10%	1 dog	12.5%	yes	yes	33%	33%
Need for ASM	67%	80%	86%	all dogs	70%	75%	50%	75%	33%
QoL dog	7.4 ± 2.8	7.2 ± 3.6		8.5 ± 2.1	6.3 ± 3.6	7.3 ± 3.6	5.3 ± 2.1	7.5 ± 2.0	9.6 ± 0.5
QoL owner	6.7 ± 3.0	6.2 ± 2.7		8.0 ± 2.8	4.6 ± 4.0	7.0 ± 3.4	4.7 ± 2.1	6.5 ± 3.2	9.4 ± 0.7

## Results

### Prevalence

**Figure 1:**
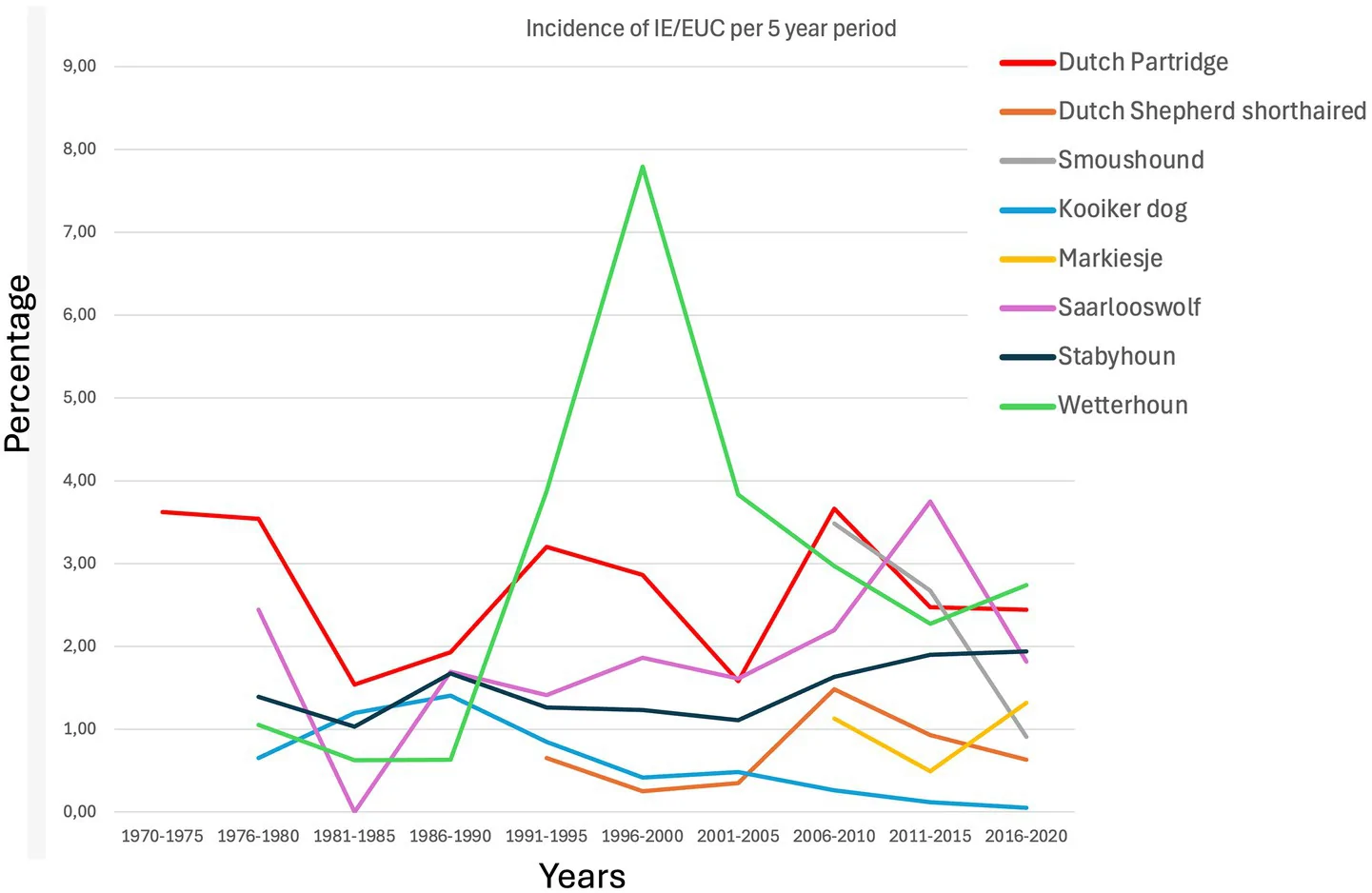
Incidence of IE/EUC per five year period.

The time period used to calculate prevalence varied by breed (Table 1). The Partridge dog breeders’ association had the most completed registrations, over a 53-year period. Overall prevalence was highest for the Wetterhoun at 2.8%, followed by the Partridge dog at 2.6%, with the lowest for the longhaired Dutch Shepherds at 0.4%, the Kooikerdog at 0.4%, and the Schapendoes at 0.2% (Table 1). For the breed clubs with the longest time period, the incidence per 5-year period could be calculated (Figure 1). The incidence of IE/EUC cases gradually increased for the Stabyhound and Wetterhoun. It decreased for the Dutch Shepherd, Smoushound, Kooikerdog and Saarloos Wolfdog. The decrease was most pronounced for the Kooikerdog, with only 0.05% in the last analysed period (Figure 1).

### Phenotypic characterisation

A total of 49 dogs were seen at one of the three clinics, and all owners completed the survey. With the help of the breeder’s associations, another 269 responses were obtained. 119 responses were removed due to missing mandatory data, and 50 dogs were excluded for failing to meet the inclusion/exclusion criteria. This resulted in 118 responses: 57 Dutch Partridge dogs, 5 Dutch Shepherds, 14 Kooiker dogs, 2 Markiesje dogs, 10 Saarloos Wolfdogs, 4 Schapendoes dogs, 4 Dutch Smoushounds, 13 Stabyhouns, and 9 Wetterhouns. For all breeds, responses came only from Dutch owners, except for the Dutch Partridge dog and the Kooiker dog. For these two breeds, the collected information came from several other Northern European countries.

### Dutch Partridge dog

#### Signalment

A total of 57 Dutch Partridge dogs were available for analysis. Thirty-three were male (58%), 24 female (42%). Thirty-five out of 57 dogs were no longer alive (62%), in 17 dogs, their death was related to the IE (49%). The mean age of death was 9.5 ± 3.5 years (median 10 years, range 1–14 years). The age of death was significantly lower when the cause of death was epilepsy (*p* < 0.010). Thirty-five dogs had reported kinship with another dog with epilepsy (61%). This kinship consisted of littermates (*n* = 16), parents (*n* = 6), half-brothers or -sisters (*n* = 13), children (n = 1), grandparents (*n* = 4) or uncles/aunts (*n* = 5). In some dogs, there were several kinship relationships, such as a half-brother and a grandparent. There was no apparent difference in character of the dogs; most were described as vivid or cheerful, nor was there a relation for vaccination status, anti-ectoparasitic or anthelmintic treatments.

#### Diagnostics

The majority of these dogs were seen by a general practitioner (86%), with 14% seen by a diplomate of the European College of Veterinary Neurology (ECVN). In 28 dogs, a TIER I level of confidence was reached; in two, a TIER II; and the remaining dogs did not reach these levels and were classified as TIER 0.

#### Numbers, severity and triggers

The mean AoO was 44 ± 23 months (median 45 months, range 6–107 months) for all 57 included dogs. Complete information was only available for 49 of 57 dogs. Thirty-three out of 49 dogs had experienced ≥10 seizures (67%). GTCS were observed in 47 of 57 dogs (82%); three of 57 had only FS (5%); and seven of 57 had a combination of GTCS and FS (14%). The mean severity of GTCS was 6.9 ± 2.6 (median 7, range 0–10) and of FS 6 ± 2.9 (median 7, range 0–10).

Nineteen owners of 49 could recognise one or more triggers before the seizure (40%). These triggers included stress (*n* = 9), weather influences (*n* = 1), visitors (*n* = 1), a specific season of the year (*n* = 1), loud noises (*n* = 2), a specific part of the day (*n* = 1), excitement (*n* = 2), sleep (*n* = 1), firework (*n* = 1) and anti-ectoparasitics (*n* = 2).

#### Pre-ictal phase

Thirty owners of 49 recognised a pre-ictal phase in their dog (61%). Pre-ictal signs included salivating (10%), restlessness (30%), clinginess (48%), aggression (2%) and a combination of acting differently or a strange look (45%). The mean length of the pre-ictal phase was 3,8 ± 12,9 h (median 5 min, range 1 min – 2 days). Twenty-eight owners of 57 were never able to predict an upcoming seizure (49%), 25 could occasionally predict a seizure (44%), and four owners were always able to predict an upcoming seizure (7%).

#### Ictus

Twenty-five dogs out 49 had seizures while sleeping (51%), 15 while resting (31%), three during a walk outside (6%), two while playing (4%), and one of 49 while waking up (2%). In three dogs, the circumstances before the seizures were unknown (6%). Ictal signs were reported in 49 of 57 dogs. The most common signs during the ictus were lying on one side (86%), cycling leg movements (82%), salivation (73%) and falling over (71%). Other ictal signs are shown in Table 3. The mean duration of the average ictal phase was 5.5 ± 3.6 min (median 5 min, range 0.5–15 min), the shortest was 3.2 ± 2.2 min (median 3 min, range 18 s-10 min) and the longest was 6.9 ± 3.9 min (median 6 min, range 1–15 min).

**Table 3:** Reported ictal signs in the Dutch Partridge dog.

Ictal signs	Number of dogs/total number	Percentage (%)
Cramping neck and legs	34/49	69%
Falling over	35/49	71%
Laying on one side	42/49	86%
Cycling motions legs	40/49	82%
Turning of the head	16/49	33%
Chewing movements	22/49	45%
Contraction facial muscles	27/49	55%
Urinating	30/49	61%
Defecating	8/49	16%
Salivating	36/49	73%
Pupil dilatation	34/49	69%
Walking in circles	15/49	31%
Gazing	21/49	43%
Clinginess	7/49	14%
Bumping into objects	23/49	47%
Temporary loss of vision	12/49	24%
Anxiousness	13/49	27%
Aggression	1/49	2%

Out of 57 dogs, 28 were experiencing CS (49%) and 12 were experiencing SE (21%). There was a significant association between the occurrence of CS and/or SE and the age at death (*p* = 0.001) and between the occurrence of CS and/or SE and the cause of death (*p* = 0.002). For one dog, it was reported that it died during an SE. In 43 out of 49 dogs, seizures had the same presentation (88%). Six out of 29 dogs showed differences between their left and right sides of the body during the seizure (12%). Two out of 49 dogs showed abnormal behaviour between seizures (4%), such as being anxious (*n* = 1) and acting apathetic (*n* = 1). Six out of 40 owners were able to shorten the seizure (15%). This could be done by talking to the dog or calling their name (*n* = 4), darkening the room (*n* = 2), or petting the dog (*n* = 1). The average frequency of seizures for dogs suffering from IE for at least 1 year was 6.8 ± 11.3 seizures in 12 months.

#### Post-ictal phase

Thirty-three of 49 dogs showed a post-ictal phase (67%). Post-ictal signs seen by the owners include fatigue (67%), walking aimlessly (43%) and drinking directly after the seizure (35%; see also Appendix 3). The mean length of the post-ictus was 8.9 ± 21.8 h (median 30 min, range 3–5,000 min). Immediately after the ictus, 41 dogs (84%) were responsive to their owner. Other post-ictal signs are shown in Table 4.

**Table 4:** Reported post-ictal signs in the Dutch Partridge dog.

Post-ictal signs	Number of dogs/total number	Percentage (%)
Fatigue	33/49	67%
Walking aimlessly	21/49	43%
Blindness	7/49	14%
Drinking directly	17/49	35%
Eating directly	7/49	14%
Wanting to go for a walk	13/49	27%
Doing nothing, laying down	3/49	6%
Vomiting	1/49	2%
Aggression	1/49	2%
Other	12/49	24%

#### ASM

A total of 38 of 57 dogs (67%) were being treated with an ASM. Thirty-three were treated with phenobarbital (PB; 87%), nine with potassium bromide (KBr; 24%), one with levetiracetam (LEV; 3%), seven with imepitoin (18%) and one with gabapentin (GAB; 3%).

Eleven dogs used a combination of ASMs (28%). The most common combination was PB and KBr (*n* = 5). Twenty-eight dogs experienced side effects (74%): increased appetite (54%) and lethargy (53%) were the most common. Other side effects are shown in Table 5. Thirty out of 48 owners had emergency medication available (63%), comprising rectal diazepam (DZP; *n* = 27), LEV (*n* = 3), and midazolam nasal spray (*n* = 1). Eight dogs were using alternative therapy (14%). This included cannabidiol (CBD) or tetrahydrocannabinol (THC) oil (*n* = 6), a special, not specified, diet (*n* = 2), cuprum sulphuricum D7 (*n* = 1), and cell salts (*n* = 1). Three dogs were using a combination of these alternative therapies. The average time from the first seizure to starting medical treatment was 10 ± 25 months (median 1 month, range 0–121 months).

**Table 5:** Anti-seizure medication (ASM) side-effects in the Dutch Partridge dog.

Side-effects	Number of dogs/total number	Percentage (%)
Lethargy	15/28	53%
Sleeping more	9/28	32%
Restlessness	3/28	11%
Ataxia	8/28	29%
Muscle weakness	6/28	21%
Increased appetite	15/28	54%
Weight gain	8/28	29%
Vomiting	1/28	4%
Polydipsia	10/28	36%
Polyuria	8/28	29%
Coughing	1/28	4%

#### QoL and the worst phase

The mean QoL score for the dog was 7.4 ± 2.8 (median 8, range 1–10) and for the owner 6.7 ± 3.0 (median 8, range 1–10). A significant association was found between the occurrence of CS and/or SE and the dog’s QoL score (*p* = 0.014). Thirty-six of 57 owners identified the ictus as the worst phase for their dog (64%), nine the post-ictus (16%), and one the pre-ictus (2%). Ten owners could not identify a specific worst phase (18%). No significant association was found between the ictus being the worst phase and the occurrence of CS and/or SE.

### Dutch Shepherd

#### Signalment

Only five Dutch Shepherds with IE were included in this study, three males and two females. For phenotypic characterisation, no difference was made between short, rough, or longhaired variants. Three of these dogs were still alive at the time of this study. The other two dogs died for reasons related to IE. They died at the ages of 1 and 6 years. For one dog, it was reported that some littermates and a parent were also diagnosed with epilepsy. Two dogs were described by their owner as vivid and cheerful, with stable characters.

#### Diagnostics

Four dogs were seen by a general practitioner, one by a diplomate ECVN. Two dogs had reached a TIER I confidence level, three TIER 0.

#### Numbers, severity and triggers

The mean AoO was 46 ± 22 months (median 51 months, range 16–65 months). Two of five dogs had experienced ≥10 seizures. Four dogs were experiencing GTCS, and one was experiencing FS. The mean severity of GTCS was 8.8 ± 1.3 (median 9, range 7–10), and of FS 4.5 ± 2.1 (median 4.5, range 3–6). One owner reported stress as a trigger for the seizures.

#### Pre-ictal phase

One owner recognised a pre-ictal phase. In this dog, the owner noted restlessness, clinginess, acting differently, and a strange look. The average length of this phase was 12 s. Two other owners were doubtful about predicting an upcoming seizure, and the other two owners could never predict an upcoming seizure.

#### Ictus

Circumstances before the seizure were walking outside (*n* = 1) and sleeping (*n* = 1).

Ictal signs were reported in two dogs. Signs during the ictus included cramping of the neck and legs (*n* = 2), falling over (*n* = 2), lying on one side (*n* = 2), cycling leg movements (*n* = 2), chewing movements of the mouth (*n* = 1), facial muscle contractions (*n* = 1), urinating (*n* = 2), defecating (*n* = 1), pupil dilatation (*n* = 1), walking in circles (*n* = 1), gazing (*n* = 1) and bumping into objects (*n* = 2). The mean duration of the ictus was 2.5 ± 0.7 min (median 2.5 min, range 2–3 min). The shortest was 1 ± 0 min (median 1 min, no range), and the longest was 4.5 ± 2.2 min (median 4.5 min, range 3–6 min). Of the five dogs, two were experiencing CS and two were having SE. The average frequency of seizures for dogs suffering from IE for at least 1 year was 3.0 ± 3.4 seizures in 12 months.

#### Post-ictal phase

Two owners could recognise a post-ictal phase. Post-ictal signs observed by the owner included fatigue (*n* = 1), walking aimlessly (*n* = 1), drinking directly after the seizure (*n* = 1), and clinginess (*n* = 1). The average length of these phases was 15 min in both dogs.

#### ASM

Four dogs received ASM. The ASM included PB (*n* = 3), KBr (*n* = 1) and imepitoin (*n* = 2). Two dogs received a combination of ASM. All four dogs experienced side effects. These side effects were lethargy (*n* = 4), sleeping more (*n* = 1), restlessness (*n* = 1), ataxia (*n* = 1), increased appetite (*n* = 2), weight gain (*n* = 1), polydipsia (*n* = 3) and polyuria (*n* = 3). One owner had rectal DZP available as emergency medication. No dogs were treated with an alternative therapy.

#### QoL and the worst phase

The mean QoL score for the dog was 7.2 ± 3.6 (median 8, range 1–10), and for the owner 6.2 ± 2.7 (median 6, range 2–9). Of the five owners, two identified the ictus as the worst phase for the dog, two the post-ictus, and one could not distinguish a specific phase as the worst.

### Kooiker dog

#### Signalment

Fourteen Kooiker dogs were available for analysis, comprising eight males and six females. At the time of data collection, 11 dogs had died. The cause of death was IE in two dogs, whilst the others had unknown causes of death. The mean age at death was 7.4 ± 5.0 years (median 8.1 years, range 1–15 years). Six dogs were reported to be related to other dogs with epilepsy, and three had unknown kinship. Reported kinship types were uncle or aunt (*n* = 2), littermate (*n* = 1), half-sibling (*n* = 1) and grandparent (*n* = 1). Two dogs were described by their owners as vivid and cheerful, with stable characters, and two as anxious or nervous. All dogs were vaccinated and received anti-ectoparasitic and anthelmintic treatments if needed.

#### Diagnostics

Eleven of 14 dogs were seen by a general practitioner, and three by a diplomate of the ECVN. Six dogs had a TIER I level confidence, three a TIER II and five TIER 0.

#### Numbers, severity and triggers

In all 14 dogs, AoO was reported, with a mean of 38.1 ± 19.5 months (median 33.1 months, range 8–70 months). Five dogs experienced ≥10 seizures, while eight experienced <10 seizures. For one dog, the information was missing. All 14 dogs had GTCS; none had FS or a combination. These GTCS were scored by one owner as an 8. Reported triggers were stress in three dogs, a specific time of day in one, and sexual arousal in another.

#### Pre-ictal phase

In three dogs, pre-ictal signs were reported. The duration of the pre-ictal phase was recorded in two dogs, ranging from 1.5 min to 20 h. The most frequently reported signs were acting differently and a strange look. Other rarely reported signs were nausea, vomiting, salivation, restlessness, clinginess, and aggression.

#### Ictus

Information was obtained from 11 Kooiker dogs concerning the ictus. Most dogs experienced seizures while sleeping (5/11, 45.5%), followed by resting (3/11, 27.3%), at a specific time of day (3/11, 27.3%), and upon waking (1/11, 9.1%). In all dogs, neck and leg cramping and salivation were observed. The mean duration of the average ictal phase was 2.0 ± 1.2 min (median 2.0 min, range 1–4 min). The shortest was 1.3 ± 0.8 min (median 1 min, range 0.8–3 min), and the longest was 3.4 ± 1.5 min (median 3 min, range 2–6 min). CS was reported in 10/12 dogs (83.3%), and SE in 1/10 dogs (10%). One owner responded to the question about left/right difference, and no difference was reported in this dog. In 11 dogs, seizures always had the same presentation, and in 8/10 dogs the behaviour was normal between seizures. The other two dogs displayed behaviour such as restlessness, urine incontinence and being easily scared. The seizure frequency per year was 32.9 ± 46.9 (median 15.0, range 0.5–101.3).

#### Post-ictal phase

Post-ictal signs were observed in 12 dogs. In 8 dogs, mainly anxiety, clinginess, disorientation, ataxia, barking, blindness and restlessness were observed. In all dogs, fatigue, aimless walking, a lust for drinking or eating, and going out were observed. The duration of the post-ictal phase was reported in 5 dogs and averaged 4.6 ± 8.1 h (median 1 h, range 3 min to 19 h). Two dogs were responsive to their owners during the post-ictal phase, whilst one was not. Three dogs seemed to remember the attack; one did not; and one owner did not know.

#### ASM

ASM treatment was used in 12 dogs. Nine of these dogs were given PB as monotherapy, and two received PB as part of polytherapy. One dog received it in combination with LEV, and one in combination with KBr and imepitoin. Four dogs had reported side effects, with the most common being increased appetite and polydipsia. Two owners reported using emergency medication, and both used DZP rectally. Two dogs were given alternative therapy consisting of Chinese herbs and homoeopathy. The average time to start medication after the first seizure was 2.5 ± 2.6 months (median 1 month, range 0–7 months).

#### QoL and the worst phase

No information was retrieved for the QoL questions.

### Markiesje dog

#### Signalment

Two dogs were submitted, one male and one female. Both dogs were alive at the time of this research. One dog had reported littermates with epilepsy. One dog was characterised as vivid and cheerful with a stable character, while the other was described as anxious or nervous. Both dogs were vaccinated and treated with anti-ectoparasitic and anthelmintic agents.

#### Diagnostics

Both dogs were seen by the general practitioner with a TIER I level of confidence.

#### Numbers, severity and triggers

The AoO was 27 months in one dog and 34 months in the other. Both dogs had experienced ≥10 seizures. One dog had GTCS, and the other had a combination of GTCS and FS. The mean severity of GTCS was 4.5 ± 5.7, and of FS 7.0 ± 0. One owner recognised multiple triggers for their dog, such as stress, walking outside, and contact with other dogs.

#### Pre-ictal phase

Both owners recognised a pre-ictal phase. Pre-ictal signs included clinginess, acting differently, or having a strange look. In one dog, the pre-ictal phase lasted 3 mins. Both owners were always able to predict an upcoming seizure.

#### Ictus

Before the seizure, in Markiesje dogs, one was resting (*n* = 1), and one was playing (*n* = 1). Both owners reported ictal signs, including cramping of the neck and legs (*n* = 2), falling over (*n* = 1), lying on one side (*n* = 2), cycling leg movements (*n* = 1), facial muscle contractions (*n* = 2), salivation (*n* = 2), pupil dilatation (*n* = 2), gazing (*n* = 2) and anxiety (*n* = 1). The average duration of the ictus was 5 mins, with the shortest ictus lasting 2 mins and the longest 8 mins. No Markiesje dogs experienced CS, but one experienced SE once. The average frequency of seizures for both dogs was 9.0 and 10.0 seizures in 12 months. Both dogs were suffering from IE for more than a year.

#### Post-ictal phase

One Markiesje dog showed post-ictal signs. These signs were fatigue, drinking directly after the seizure and wanting to go for a walk. The average length of the pre-ictus was 1 min. Directly after the ictus, both Markiesje dogs were responsive to their owner.

#### ASM

Both dogs received an ASM; one received PB and the other imepitoin. One dog experienced side effects of the ASM, including increased sleep and polyuria. One owner used rectal DZP as emergency medication. No dogs were receiving alternative therapy. One dog started medication after the first seizure; the other after 11 months.

#### QoL and the worst phase

The mean QoL-score of the dog was 8.5 ± 2.1 and of the owner 8.0 ± 2.8. Both owners mentioned the ictus as worst phase for the dog.

### Saarloos Wolfdog

#### Signalment

Ten Saarloos Wolfdogs were included, comprising seven males and three females. At the time of data collection, five dogs were reported to be deceased. The cause of death was IE in four dogs. The mean age at death was 5.2 ± 4.0 years (median 4 years, range 2–12 years). Six of the 10 dogs had other dogs with epilepsy in their family. Kinship types included littermate (*n* = 2), parent (*n* = 2), child (*n* = 1), grandparent (*n* = 1) and uncle or aunt (*n* = 1). Six dogs were described as vivid and cheerful, with stable characters, and one as nervous. Five of the 10 dogs received vaccination, anthelmintic drugs and anti-ectoparasitic drugs.

#### Diagnostics

Six out of 10 dogs were seen by a general practitioner, and four by a diplomate of the ECVN. In five dogs, TIER I confidence was achieved, and in two, TIER II. Three dogs were classified as TIER 0. Additional diagnostics included cardiac ultrasound in two dogs and an electrocardiogram (ECG) in one dog.

#### Numbers, severity and triggers

The average AoO was 40.7 ± 21.9 months (median 43.5 months, range 12–66 months). Four dogs experienced ≥10 seizures, and six had <10. Seizure types ranged from FS and GTCS (2/10) to GTCS alone (8/10). The severity of GTCS was scored with a mean of 7.6 ± 3.6 (median 9.5, range 0–10), and that of FS with a mean of 5.3 ± 2.5 (median 5.5, range 2–8). In four of 10 dogs, no trigger was observed by the owners. The other two owners reported stress, sexual arousal, a clear time of day, and weather as triggers.

#### Pre-ictal phase

The pre-ictal phase was observed in three dogs. All three dogs displayed unusual behaviour and a strange look. The duration of the pre-ictal phase was 1.5 min, 24 h and 36 h. Three owners could predict an impending seizure, but all others could not.

#### Ictus

Seizures occurred during rest (3/6), sleep (2/6) and walking outside (1/6). Ictal signs were reported in six dogs. All six dogs lay on one side, salivated and had cramping of the neck and legs. The mean duration of the ictal phase was 2.9 ± 3.0 min (median 2 min, range 0.2–8 min). The shortest and longest durations were 1.5 ± 1.3 min (median 1.0 min, range 0.16–4 min) and 4.4 ± 3.4 min (median 4.0 min, range 0.33–10 min), respectively. The seizures had the same presentation in all six dogs. Five out of eight dogs had CS and 1/8 had SE. A left/right difference was observed in two dogs, while three owners did not notice it and one did not know. Four dogs behaved normally between seizures, and two did not. They displayed the following behaviours: continuous food-seeking, insecurity, and unawareness of their surroundings. Four owners were able to shorten the seizure. The mean frequency of seizures per year was 21.1 ± 19.1 (median 19.9, range 0.7–44.2).

#### Post-ictal phase

Post-ictal signs were observed in six dogs. The most commonly reported signs were walking aimlessly (83.3%), fatigue (66.7%), and drinking immediately afterwards (66.7%). The mean duration of this phase was 35.3 ± 74.2 h (median 1.0 h, range 5 min to 7 days). Five of seven dogs responded to their owners, while two did not. Two of six dogs appeared to remember the seizure, three did not, and one owner did not know.

#### ASM

Seven dogs received ASM treatment. All seven dogs received PB, with two receiving it as monotherapy and five in combination with one or more other ASMs. These included KBr (3/7) and imepitoin (3/7). Three of six dogs received emergency medication. One received DZP rectally, one LEV, and the third both. Side effects were reported in six of seven dogs, with the most common being lethargy (66.7%), muscle weakness (66.7%), and increased appetite (66.7%). Two of seven dogs also received alternative therapy, consisting of Medium Chain Triglyceride (MCT) oil and a BARF special diet. Three owners started medical management immediately after the first seizure, while one owner waited 3 months and another 35 months.

#### QoL and the worst phase

The QoL questions were answered by seven owners. The dog’s QoL was scored with a mean of 6.3 ± 3.6 (median 8, range 1–10), and the owner’s QoL with a mean of 4.6 ± 4.0 (median 3, range 1–10). The ictus was rated the worst phase for the dog by 4/7 owners, and the pre-ictal phase by three owners.

### Schapendoes dog

#### Signalment

Four Schapendoes with IE were included in this study, all of them were males. Two dogs had died at the time of this study. One of them died due to IE. They died at ages of 6 and 10 years. Out of three dogs, one had reported kinship with another dog with epilepsy. This kinship included littermates, grandparents and aunts/uncles. Three dogs were characterised by their owners as vivid or cheerful, with stable characters, and one as nervous or anxious. All dogs were vaccinated and treated with antiparasitic and anthelmintic agents.

#### Diagnostics

Of the four dogs, one was seen by the general practitioner and the other three by a diplomate of the ECVN. In two dogs, a TIER I level of confidence was reached, and in two, a TIER II level of confidence was reached.

#### Numbers, severity and triggers

The mean AoO was 59 ± 18 months (median 63 months, range 36–74 months). Two dogs had ≥10 seizures. All four dogs were experiencing GTCS. The mean severity of these seizures was 8.3 ± 1.5 (median 8, range 7–10). Two owners recognised a trigger, including a specific time of day (*n* = 1) and holding urine or faeces (*n* = 1).

#### Pre-ictal phase

Two owners recognised a pre-ictal phase. Pre-ictal signs were restlessness (*n* = 1) and aggression (*n* = 1). One owner reported the pre-ictal phase lasting 2 h. One owner was never able to predict an upcoming seizure; two owners occasionally could; and one owner could always predict one.

#### Ictus

Two Schapendoes dogs experienced seizures while sleeping. For one dog, the circumstances preceding the seizures were unknown. Ictal signs were reported in three dogs. Signs observed during the ictus included cramping of the neck and legs (*n* = 3), falling over (*n* = 1), lying on one side (*n* = 2), cycling leg movements (*n* = 2), chewing movements of the mouth (*n* = 2), facial muscle contractions (*n* = 1), salivation (*n* = 2), walking in circles (*n* = 1) and barking (*n* = 1). The mean duration of the average ictus was 3.0 ± 1.7 min (median 2 min, range 2–5 min). The shortest was 1.3 ± 0.6 min (median 1 min, range 1–2 min), and the longest was 4 min in all dogs. Out of three dogs, one was experiencing CS and two were having SE. Three Schapendoes dogs showed uniform seizures and normal behaviour between seizures. One owner was able to shorten the seizure by petting/talking to their dog. The average frequency of seizures for dogs suffering from IE for at least 1 year was 2.1 ± 2.0 seizures in 12 months.

#### Post-ictal phase

Two owners recognised a post-ictal phase. Post-ictal signs observed were fatigue (*n* = 1), walking aimlessly (*n* = 2), drinking immediately after the seizure (*n* = 1) and wanting to go for a walk (*n* = 1). The average duration of the post-ictal phase in both dogs was 1 h. Immediately after the ictus, three dogs were responsive to their owners.

#### ASM

Three dogs received an ASM: PB (*n* = 1), KBr (*n* = 1), LEV (*n* = 1) and imepitoin (*n* = 2). Two dogs received a combination of ASMs. All dogs experienced side effects from the medication. These side effects were lethargy (*n* = 1), increased sleep (*n* = 1), ataxia (*n* = 1), muscle weakness (*n* = 2), increased appetite (*n* = 2), weight gain (*n* = 2), vomiting (*n* = 1) and coughing (*n* = 1). One owner had rectal DZP available as emergency medication. No dogs were treated with an alternative therapy.

#### QoL and the worst phase

The mean QoL score for the dog was 7.3 ± 3.6 (median 8.5, range 2–10), and for the owner 7 ± 3.4 (median 8.5, range 2–9). One owner identified the ictus as the worst phase for their dog, one the post-ictus, and two could not distinguish a specific phase as worst.

### Dutch Smoushound

#### Signalment

Four Dutch Smoushounds were included in this study, two males and two females. Two dogs were no longer alive at the time of the study. In both cases, their deaths were related to IE, and they died at the ages of 2 and 5 years. Of the four dogs, two had littermates with epilepsy. Two dogs were described by their owners as vivid and cheerful, with a stable character, and one as nervous or anxious. For two dogs, their vaccination schedules were up to date. Three dogs were being treated with anti-ectoparasitics, and one with anthelmintics.

#### Diagnostics

One dog was seen by the general practitioner, and three by a diplomate of the ECVN. In one dog, a TIER I level of confidence was reached, and in three, a TIER II.

#### Numbers, severity and triggers

The mean AoO was 24 ± 32.9 months (median 10 months, range 3–73). One dog had experienced ≥10 seizures. Three dogs had GTCS, and one dog was experiencing a combination of GTCS and FS. The mean severity of GTCS seizures was 5.0 ± 3.6 (median 5, range 1–9). The severity of FS was scored as 10. Two owners could recognise a trigger for the seizures, such as stress (*n* = 1) and gluten sensitivity (*n* = 1).

#### Pre-ictal phase

In no dog could a pre-ictal phase be recognised. In addition, none of the owners could predict an upcoming seizure.

#### Ictus

Before the seizure, the dogs were sleeping (*n* = 2) or resting (*n* = 1). Ictal signs were reported in three dogs. These signs consisted of cramping of the neck and legs (*n* = 1), falling over (*n* = 2), cycling leg movements (*n* = 3), chewing movements of the mouth (*n* = 2), facial muscle contractions (*n* = 1), urinating (*n* = 1), defecating (*n* = 2), salivating (*n* = 1), pupil dilatation (*n* = 1) and anxiousness (*n* = 1). One ictal duration of 30 min was removed due to unreliability. The mean duration of the average ictus was 6.7 ± 7.4 min (median 4 min, range 1–15 min). The shortest was 2.3 ± 2.3 min (median 1 min, range 1–5 min), and the longest was 3.5 ± 3.5 min (median 3.5 min, range 1–6 min). Three dogs experienced CS, and two dogs experienced SE. In all four dogs, seizures had the same presentation. There were no dogs with a left–right difference during the ictus, and no owners were able to shorten the seizures. The average frequency of seizures, for dogs suffering from IE for at least 1 year, was 1.1 ± 0.6 seizures in 12 months.

#### Post-ictal phase

None of the owners recognised a post-ictal phase. Immediately after the seizure, three dogs were responsive to their owners.

#### ASM

Two dogs received PB as ASM. The other two dogs did not receive any ASM. Both dogs receiving ASM experienced side effects. These side-effects included lethargy (*n* = 2), sleeping more (*n* = 2), muscle weakness (*n* = 1), increased appetite (*n* = 1), polydipsia (*n* = 1) and skin rash (*n* = 1). None of the owners had emergency medication available. One dog was being treated with alternative therapy, consisting of a combination of phytotherapy, CBD- and THC-oil, a special diet and music therapy.

#### QoL and the worst phase

The mean QoL score for the dog was 5.3 ± 2.1 (median 6, range 3–7), and for the owner 4.7 ± 2.1 (median 4, range 3–7). Two owners described the ictus as the worst phase for their dog, and one owner described the post-ictus.

### Stabyhoun

#### Signalment

Thirteen Stabyhouns were included in the study. Eight dogs were male (61.5%), and five were female (38.5%). Four dogs were deceased (30.8%) at the time of data collection, of which two deaths were related to IE. The mean age at death was 7.8 ± 4.5 years (median 6.5 years, range 4–14 years). Five out of 13 dogs had a relative with epilepsy, two did not, and for three it was unknown. Types of kinship reported were littermate (*n* = 4), parent (*n* = 1), half-sibling (*n* = 4), or offspring (*n* = 1). Six dogs were described by their owners as vivid and cheerful with a stable character, and three as nervous or anxious. Eight out of 13 dogs were vaccinated, 5/7 received anthelmintic drugs, and 7/8 received anti-ectoparasitic treatments.

#### Diagnostics

Eight out of 13 were seen by the general practitioner (61.5%), and five by a diplomate of the ECVN (38.5%). In seven dogs, a TIER I, and in five a TIER II, and in one a TIER 0 level of confidence was reached.

#### Numbers, severity and triggers

The average AoO was 39.4 ± 17.7 months (median 35 months, range 12–76 months). Five of 13 dogs experienced ≥10 seizures (38.5%), and 8/13 had <10 seizures (61.5%). GTCS were reported in 12 dogs (92.3%), and FS in one dog (7.7%).

The severity of GTCS was 6.5 ± 2.0 (median 6.5, range 3–10), and of FS 4.0 ± 2.7 (median 4, range 1–9). Five of eight owners did not recognise a trigger for the seizures, while the other three reported stress (*n* = 1), a specific time of day (*n* = 1), and dog daycare (*n* = 1).

#### Pre-ictal phase

A pre-ictal phase was recognised in three dogs, and the most common sign was restlessness (57.1%). The duration was reported in two dogs and was 3 min and 10 min. Thirteen owners rated the predictability of the ictus: 4 were unable to predict, and the others were able to predict a coming seizure, though this varied by dog.

#### Ictus

Circumstances were reported in seven dogs. The onset of seizures most commonly occurred during sleep (3/7), followed by waking (1/7) and resting (1/7). One owner reported variability in circumstances. Ictal signs were reported in eight dogs, and the most frequently reported signs were lying on one side (87.5%), cycling leg movements (87.5%) and salivation (87.5%). The mean duration of the ictus was 2.9 ± 2.1 min (median 2.5 min, range 1–7 min). The shortest was 1.6 ± 1.2 min (median 1.0 min, range 0.5–4.0 min), and the longest was 3.3 ± 1.8 min (median 3.0 min, range 1–6 min). Eight out of 13 dogs experienced CS (61.5%), and 4/12 dogs SE (33.3%). Three owners reported a difference between left and right, while five did not know. All eight owners reported that every seizure had the same presentation. Six of the eight dogs behaved normally between seizures. Two showed different behaviour from before, such as being calmer and slower. Four owners were unable to shorten the seizure, and two reported they did not know. The average seizure frequency per year was 6.3 ± 8.3 (median 2.9, range 0.1–24.0).

#### Post-ictal phase

Eight dogs showed post-ictal signs. The most common sign was aimless walking (75%). Other rarely reported signs were anxiety, clinginess, disorientation, ataxia, barking, blindness, a lust for drinking or eating, and going out. The mean duration of the post-ictal phase was 51.6 ± 102.3 min (median 8.5 min, range 2 min to 5 h). During the post-ictal phase, three of four dogs were responsive to their owners. One of eight dogs seemed to remember the seizure, 5/8 did not, and one owner did not know.

#### ASM

A total of nine dogs were treated with an ASM (75%). Seven received phenobarbital (PB), five also with potassium bromide (KBr), two only imepitoin and two also with levetiracetam (LEV). Hence, seven of these dogs received a combination of more than one ASM. Side-effects from the ASM occurred in 8 dogs (88.8%). Six of 10 owners had emergency medication available. Types of emergency medication were DZP rectally (4/6), levetiracetam (LEV; 1/6) and a combination of DZP rectally, nasal spray midazolam and LEV (1/6). Three of 12 dogs also received alternative therapy (25%), including cannabidiol (CBD) oil (*n* = 2) and homoeopathy (*n* = 1). The average time owners waited to start medical treatment after the first seizure was 2.2 ± 2.4 months (median 1.5 months, range 0–6 months).

#### QoL and the worst phase

The QoL of the dog and the owner were scored with means of 7.5 ± 2.0 (median 8, range 4–10) and 6.5 ± 3.2 (median 8, range 1–10), respectively. Six out of 11 owners identified the ictus as the worst phase for the dog (54.5%), three identified the post-ictus (27.3%), and two identified no specific phase (18.2%).

### Wetterhoun

#### Signalment

Nine Wetterhoun’s were included in the study. There was no sex difference (five males and four females). At the time of data collection, four were reported as deceased. Three dogs died for reasons unrelated to IE, and in the fourth dog the cause was not reported. The mean age at death was 8.8 ± 4.0 years (median 10 years, range 3–12 years). Seven of the nine dogs were reported to be related to another dog with epilepsy. Types of kinship were littermate (*n* = 5), parent (*n* = 1) and half-sibling (*n* = 3). Six dogs were described by their owners as vivid and cheerful with a stable character, and three as nervous or anxious. Nine dogs were vaccinated, six received anthelmintic drugs, and eight received anti-ectoparasitic treatments.

#### Diagnostics

Eight out of nine dogs were seen by a general practitioner, and one by an ECVN diplomate. In three dogs, TIER I confidence was reached; in one, TIER II; in all others, TIER 0.

#### Numbers, severity and triggers

The mean AoO of the first seizure was 29.8 ± 17.6 months (median 27.5 months, range 9–69 months). Five dogs experienced ≥10 seizures, and four experienced <10 seizures. Six dogs showed GTCS, one showed FS, and two showed both. GTCS were scored on average 5.0 ± 4.0 (median 7, range 0–10), and FS 4.6 ± 3.8 (median 7, range 0–8). Triggers were reported in 3/9 dogs, including stress (*n* = 1), hormonal influence (*n* = 1), and vaccination (*n* = 1).

#### Pre-ictal phase

A pre-ictal phase was reported in seven dogs. The average duration was 7.0 ± 4.2 min (median 8.5 min, range 1–10 min), and the most common signs during the pre-ictal phase were clinginess (57.1%) and acting differently/strange-looking eyes (42.9%). Other rarely reported signs were nausea, vomiting, salivation, restlessness, clinginess, aggression, acting differently and having a strange look. The predictability of the seizures was scored by 9 owners; 5 could not predict an impending seizure, and 4 could, though this varied by dog.

#### Ictus

Five out of eight dogs had seizures while walking outside, two while resting and two while sleeping. Signs during the ictal phase were reported in nine dogs. The most common signs were salivating (100%), lying on one side (77.8%) and cramping of the neck and legs (77.8%). The mean duration of the ictus was 3.7 ± 1.2 min (median 3 min, range 3–5 min). The longest and shortest durations were 1.7 ± 0.6 min (median 2 min, range 1–2 min) and 5.7 ± 0.6 min (median 6 min, range 5–6 min), respectively. On 2/9 dogs, CS were reported, and on 3/9 dogs, SE. In seven out of nine dogs, the seizure always had the same presentation, and eight dogs behaved normally between seizures. One dog was more anxious than before. There was no left/right difference in five dogs, and four owners did not know. Two out of six owners could shorten the seizure by calming the dog or putting a cloth over the head. Three owners were not able to shorten the seizure. The average seizure frequency per year is 2.2 ± 1.9 (median 2.2, range 0.4–6.2).

#### Post-ictal phase

Six dogs displayed post-ictal signs. The most frequently reported sign was fatigue (88.9%). Other reported signs included anxiety, clinginess, disorientation, ataxia, barking, blindness, restlessness, a lust for drinking or eating, and going out. The mean duration of the post-ictal phase was 53.3 ± 31.4 min (median 45 min, range 20–90 min). During this phase, seven dogs were responsive to their owners, while one was not. One dog seemed to remember the seizure, while three did not seem to, and five owners did not know.

#### ASM

Three dogs were medicated with ASM. Two dogs received PB as monotherapy, and one received a combination of imepitoin and LEV. Side effects occurred in two dogs. Both had increased appetite, and one also had weight gain and polydipsia. DZP rectally was used as emergency medication in 3/5 dogs, while two did not have any emergency medication. One dog was given a special diet and drops as alternative medication. One owner started medication immediately after the first seizure, while another started after 38 months.

#### QoL and the worst phase

The owners rated the dog’s QoL with a mean of 9.6 ± 0.5 (median 10, range 9–10) and their own mean QoL with 9.4 ± 0.7 (median 10, range 8–10). Six out of nine owners reported the ictus as the worst phase for the dog, and three reported no specific phase. There was a weak but significant association between owners rating the ictus as the worst phase and the occurrence of CS and/or SE (*p* = 0.047).

## Discussion

IE/EUC, either GTCS, FS, or combinations, were reported in all nine Dutch breeds. The overall prevalence varied by breed, with the Dutch Partridge dog, Saarloos Wolfdog, Stabyhoun, and Wetterhoun reporting higher percentages than the general dog population, which is 0.6–0.75% (7, 28). If a breed experiences a high incidence of epilepsy, a breed club may take proactive measures. All Dutch breed clubs do this, but it is challenging to manage relatives of IE/EUC cases if the current breeding stock is already small. The recurrence of the phenotype in related individuals is suggestive of a hereditary basis in at least these families where this recurrence is observed. Based on the number of registered dogs (source: www.dutchdogdata.nl) and the estimated number of dogs alive (source: the Dutch breed clubs), the Dutch Kooiker dog has the largest population by number. When the Kooiker breeders faced a number of other diseases (10, 22), for which the causative mutation had not yet been identified, they began using risk calculation (9). Based on the results of this study, they were able to reduce the incidence and overall prevalence of IE/EUC. A similar strategy has been used successfully by breeders of the Smoushound, Dutch Shepherd, and Saarloos Wolfdog. However, this could not be applied by all breed clubs. The Markiesje dog, Saarloos Wolfdog, Smoushound, Stabyhoun and Wetterhoun have very low numbers, which is a major challenge for sustainable breeding. To overcome problems, breeders of the Markiesje dog, Saarloos Wolfdog, Stabyhoun and Wetterhoun use outcrossing to improve the breeds (source: https://www.markiesjesvereniging.nl/; https://avls.nl/; www.nsvw.nl).

Earlier, in 2017, Mandigers reported prevalence data of the nine breeds (13). The number of cases has, except for the Wetterhoun and Markiesje, slowly declined. The numbers in this study differ slightly, except for the two breeds mentioned. Mandigers used only data from the breed clubs, whereas this study also includes cases that were not reported to or made known to the breed clubs. But regardless of the overall prevalence, the decline in case frequency across several breeds is encouraging, demonstrating that risk calculations can even help in small-breed populations.

The phenotypic characteristics of IE/EUC in these nine breeds are comparable with those of several breeds in which IE/EUC has been described earlier (2). The most notable difference is the reported high QoL scores for both dog and owner for eight of these breeds compared to other breeds such as the Irish Setter, Rottweiler, Great Swiss, Border Collie and Australian Shepherd (1, 5, 8, 25, 26, 29, 30). The only exception is the QoL scores for the Smoushound. They are low, even though the number of seizures is lower than in the other breeds. However, the seizure duration is longer than in most breeds. It must be noted that only four Smoushound owners participated in this study.

Except for the Dutch Partridge dog, the phenotypic characteristics of these breeds had not yet been reported. The Dutch Partridge dog has been described earlier in a study by Hamers et al. (1), but that study counted only 14 Dutch Partridge dogs. In this study, 57 dogs could be included.

A slightly worrying observation is the late AoO for most of the nine breeds. Most breed clubs allow the use of bitches from 24 months, and in some cases, this is raised to 36 months for sires. This allows breeders to make choices based on a longer study period and, in some cases, to avoid breeding with dogs that may develop IE/EUC. This applies, for instance, to the Border Collie and Australian Shepherd, which have a low AoO (1, 8, 26, 29). However, in this study, 6 of 9 breeds had an average AoO greater than 3 years. This complicates matters for the breeds if they start using their breeding stock at a young age, as this introduces the risk of breeding a dog that develops IE/EUC after being used.

As in all earlier studies, death related to IE/EUC is associated with the presence of CS and SE, and a high frequency of seizures. Except for the Saarloos Wolfdog and Kooiker dog, the average number of seizures per year was rather low. Both the Kooiker dog and Saarloos Wolfdog had very high numbers, and this was clearly related to an (elected) earlier death. In both breeds, the cases that died from epilepsy used one of two ASMs.

Stress or other triggers were present in all breeds but were not observed in all dogs, as reported earlier (31, 32).

Currently, a number of genetic studies are ongoing in these nine breeds, and the findings of this study may help identify causative mutations and/or genetic risk factors, as described earlier by Beckers et al. (24) in the Dutch Partridge dog.

This study has some clear limitations. First, the sample size is very small for 4 breeds, so the phenotypic characteristics must be interpreted with caution. However, the semiology of the reported IE/EUC in these breeds is comparable with that of most other breeds (1, 2), except for the mentioned differences. Secondly, despite our efforts, it is very likely that not all owners of dogs that suffered from IE/EUC could be reached. Thirdly, we used a slightly different approach compared to the recently proposed survey technique (33). However, the final phenotypic classification is comparable. Fourth, ideally, all included dogs would at least have reached TIER I, but this is more wishful thinking than reality. Even in the recently published genetic study investigating IE in the Dutch Partridge dog, only 5 of 16 dogs reached a TIER I or TIER II level of confidence. Reported reasons include, among others, some owners not wishing to have their dogs taken to the veterinarian for a blood sample or more, some owners simply lacking the money, and, sadly, some dogs refusing to be examined. Fifth, although the possible influence of external seizure-precipitating factors (triggers) was addressed in the survey, the number of included dogs is limited to allow a proper analysis. Only, stress was the most commonly noted trigger across most breeds. Stress as a precipitating factor has been reported earlier (31, 32). External factors can influence the expression of IE in breeds, as demonstrated in the Border Collie (31). In the Border Collie, a very vivid and sensitive breed, the relationship between the epileptic dog and its environment seems present (31), although it was not observed in another vivid and sensitive breed, the Labradoodle (6). Environmental factors are most likely to influence the expression of IE and therefore need to be addressed in any future study investigating IE.

Lastly, although diet and dietary change were addressed in the survey, the information provided was insufficient for a proper analysis. Diets and their exact composition may also play a role in the expression and management of IE, as recently demonstrated (34). Including this in further studies seems to be warranted.

## Conclusion

This study described the phenotypic characterisation and prevalence of IE/EUC in the nine Dutch dog breeds. Prevalence varied across the nine breeds, regardless of their population size. The phenotypic characteristics are comparable with those of other breeds, except for a late AoO in the Dutch Partridge dog (44 ± 23 months), Dutch Shepherd (46 ± 22 months), Kooiker dog (38 ± 20 months), Saarloos Wolfdog (41 ± 22 months), Schapendoes (49 ± 18 months) and Stabyhoun (39 ± 18 months). Ictus duration was high in the Dutch Smoushound (6.7 ± 7.4 min), possibly reducing the QoL score for the dog and owner, which, compared with other breeds, was previously reported as high. IE/EUC is most likely heritable in these breeds, as in earlier-reported breeds, and the breeding measures applied by the Dutch Kooiker dog and Smoushound breed clubs appear to have reduced numbers over the years. Further (epi-)genetic studies may help breeders reduce the incidence of IE/EUC in all of these breeds.

## Data Availability

The raw data supporting the conclusions of this article will be made available by the authors, without undue reservation.

## Footnotes

1 Qualtrics: https://www.qualtrics.com/.
