# Supplementary material for: Phenotypic characterisation, quality of life and prevalence of idiopathic epilepsy and epilepsy of unknown cause in the nine Dutch dog breeds
Source: Front Vet Sci. 2026 Aug 27;13:1897489. doi: 10.3389/fvets.2026.1897489 (PMC13587619; doi:10.3389/fvets.2026.1897489)
Supplement: Supplementary file 1 [file Data_Sheet_1.docx]

Epilepsieonderzoek Nederlandse rassen

Start van blok: intro

Q1
Beste eigenaar,
  Hartelijk dank voor uw bijdrage. Deze is zeer waardevol. Het doel van dit onderzoek is de epilepsie, die bij de Nederlandse hondenrassen voorkomt, beter in kaart brengen zodat we gerichter kunnen behandelen, en mogelijk beter kunnen bestrijden. Meer informatie over epilepsie kunt u vinden op de website www.veterinair-neuroloog.nl   Het invullen van de enquête duurt ongeveer 15 tot 20 minuten. Voordat u verder gaat kunt u het beste de stamboom, epilepsie dagboek en de medicatie van uw hond erbij pakken. Niet bij iedere vraag is een antwoord vereist. Bij sommige wel. Het kan zijn dat uw hond niet meer in leven is. Toch zijn uw antwoorden dan nog steeds waardevol. De vragen zijn in tegenwoordige tijd gesteld. Indien uw hond is overleden lees het dan als voltooid verleden tijd. En in de tekst spreken we steeds uw hond aan met hij/hem.   Alle gegevens worden vertrouwelijk verwerkt en worden niet gedeeld met derden tenzij u hiervoor toestemming geeft. Indien u vragen en/of opmerkingen heeft dan kunt u deze op de laatste pagina van de enquête plaatsen.
 
Misschien twijfelt u of uw hond epilepsie heeft. Kijk dan vooraf naar deze filmpjes. Zowel focale als gegeneraliseerde aanvallen zijn mogelijk. Indien u eerst meer hierover wilt lezen dan kunt u naar deze website gaan voor meer informatie: https://www.veterinair-neuroloog.nl/ziektes/epilepsie
 
Voorbeelden van focale aanvallen kunt u hier zien: https://youtu.be/AZhL9-_MvEM
 
Een voorbeeld van een zogenaamde tonisch-clonische aanval kunt u hier zien: https://youtu.be/6ticKGNgojw
 
Naast dit komt bij honden ook een zogenaamde paroxsymale dyskinesie voor. Een voorbeeld hiervan kunt u hier zien: https://youtu.be/96UMxPZH1OA
 
Het kan zijn dat u beide types bij uw hond ziet. Als er twijfel is neem dan contact met ons op.  Hartelijke dank voor het invullen van de enquête.  
dr. Paul Mandigers 
Dierenarts-specialist Veterinaire Neurologie
Expertise Centrum Genetica Diergeneeskunde
Faculteit Diergeneeskunde - Universiteit Utrecht 
& 
Evidensia Dierenziekenhuis Arnhem

Q114 Gaat u akkoord met het gebruik van uw ingevulde gegevens voor onderzoek?

- Akkoord maar u mag de gegevens NIET delen met de rasvereniging(en) (1)
- Akkoord en u mag het delen met de rasvereniging(en) (2)
- Niet akkoord (3)

Deze vraag weergeven:

If Gaat u akkoord met het gebruik van uw ingevulde gegevens voor onderzoek? = Niet akkoord

Q115 U bent niet akkoord gegaan met het gebruik van uw ingevulde gegevens, hiermee heeft u de einde van deze enquête bereikt.

Ga naar: Einde enquête Als U bent niet akkoord gegaan met het gebruik van uw ingevulde gegevens, hiermee heeft u de einde va... Weergegeven

Einde blok: intro

Start van blok: Vragen hond

Q9 Vragen over uw hond

Q47 Is uw hond nog in leven?

- Ja (1)
- Nee (2)

Deze vraag weergeven:

If Is uw hond nog in leven? = Nee

Q48 Op welke leeftijd is uw hond overleden? (in jaartallen)

________________________________________________________________

Deze vraag weergeven:

If Is uw hond nog in leven? = Nee

Q49	Wat was de oorzaak van het overlijden van uw hond?

- Ten gevolge van het feit dat hij epilepsie had (1)
- Niet gerelateerd aan de epilepsie (3)

Q17 Tot welk ras behoort uw hond?

- Smoushond (4)
- Markiesje (5)
- Wetterhoun (6)
- Stabij (7)
- Hollandse herder (8)
- Saarlooswolfshond (9)
- Kooikerhondje (10)
- Schapendoes (11)
- Drentsche Patrijshond (12)

Deze vraag weergeven:

If Tot welk ras behoort uw hond? = Hollandse herder

Q78 Welke variëteit?

- Korthaar (4)
- Langhaar (5)
- Ruwhaar (6)

| Pagina-einde |  |
| --- | --- |

Q129 Wat is de roepnaam van uw hond?

________________________________________________________________

Q18 Heeft uw hond een stamboom?

- Ja (1)
- Neen maar zijn (voor)ouders wel (2)
- Weet ik niet (4)

Q20 Indien van toepassing: Stamboomnummer (graag volledig nummer inclusief cijfers van de uitgever. Dus bijvoorbeeld NHSB 3214567)

________________________________________________________________

Q21 Chipnummer van uw hond?

________________________________________________________________

Q22 Wat is de geboortedatum van uw hond? U mag het opschrijven of zo invullen: dd/mm/jjjj

________________________________________________________________

________________________________________________________________

________________________________________________________________

________________________________________________________________

________________________________________________________________

Q23 Wat is het geslacht van uw hond?

- Mannelijk (1)
- Vrouwelijk (2)

Q24 Is uw hond gesteriliseerd of gecastreerd?

- Ja (1)
- Nee (2)

Q57 Is uw hond verwant aan andere epilepsielijders?

- Ja (1)
- Nee (2)
- Weet ik niet (3)
- Indien ja, wat is de verwantschap? (4) __________________________________________________

| Pagina-einde |  |
| --- | --- |

Einde blok: Vragen hond

Start van blok: Karakter

Q10 Het karakter van uw hond

Q35 Hoe omschrijft u het karakter van uw hond? (meerdere antwoorden zijn mogelijk)

- Levendig (1)
- Opgewekt (2)
- Kalm (3)
- Zenuwachtig (4)
- Angstig (5)
- Agressief (6)
- Overige (geef nadere toelichting) (7) __________________________________________________

Einde blok: Karakter

Start van blok: Vaccinaties / ontwormingen

Q132 Vragen over vaccinaties, anti-parasitica en anti-helmintica

Q133 Wat is van toepassing voor uw hond? Meerdere antwoorden zijn mogelijk.

- Hij krijgt zijn vaccinaties indien dat nodig is (1)
- Hij wordt regelmatig ontwormt (2)
- Hij krijgt indien nodig een vlooien en teken bestrijding (3)
- Hij wordt NIET gevaccineerd (4)
- Ik hoef hem niet te ontwormen (5)
- Ik hoef hem niet te behandelen tegen vlooien en teken (6)

Deze vraag weergeven:

If Wat is van toepassing voor uw hond? Meerdere antwoorden zijn mogelijk. = Hij krijgt indien nodig een vlooien en teken bestrijding

Q82 Wat voor vlooien en teken bestrijding geeft u uw hond?

- Ik geef hem (1) __________________________________________________

Einde blok: Vaccinaties / ontwormingen

Start van blok: Eerste aanvallen

Q11 Vragen over de (eerste) aanvallen.

Q126 Wanneer heeft u de eerste aanval bij uw hond gezien? Indien u de datum niet exact meer weet, geef dan een datum bij benadering. (dd/mm/jjjj)

________________________________________________________________

________________________________________________________________

________________________________________________________________

________________________________________________________________

________________________________________________________________

Q40 Heeft uw hond meer dan 1 aanval gehad?

- Ja (1)
- Nee (2)

Q41 Hoeveel aanvallen heeft uw hond tot op heden gehad? Vul een getal in.

________________________________________________________________

Q44
Honden kunnen diverse soorten epileptiforme aanvallen krijgen. Zowel focale als gegeneraliseerde aanvallen zijn mogelijk. Indien u eerst meer hierover wilt lezen dan kunt u naar deze website gaan voor meer informatie: https://www.veterinair-neuroloog.nl/ziektes/epilepsie
 
Voorbeelden van focale aanvallen kunt u hier zien: https://youtu.be/AZhL9-_MvEM
 
Een voorbeeld van een zogenaamde tonisch-clonische aanval kunt u hier zien: https://youtu.be/6ticKGNgojw
 
Naast dit komt bij de lhond ook een zogenaamde paroxsymale dyskinesie voor. Een voorbeeld hiervan kunt u hier zien: https://youtu.be/96UMxPZH1OA
 
Het kan zijn dat u beide types bij uw hond ziet. Als er twijfel is neem dan contact met ons op.

| Pagina-einde |  |
| --- | --- |

Q124 Wat voor type aanval(len) vertoont uw hond?

- Gegeneraliseerde tonisch-clonische aanvallen. Dit zijn aanvallen waarbij de hond in de regel op zijn zijde ligt, krampen en fietsbewegingen heeft, trekkingen met de mond, in de regel krijgt u geen contact met hem/haar en kan speeksel, urine en ontlastingsverlies optreden. (1)
- Focale aanvallen. Dit zijn, bijvoorbeeld, aanvallen waarbij slechts een deel van het lichaam de abnormaliteit laat zien. Voorbeelden zijn: trekken met de lippen, speekselen, alleen een pootje etc. (2)
- Een combinatie van zowel tonisch-clonische aanvallen en de focale aanvallen. (3)
- Hij heeft geen epilepsie maar een paroxysmale dyskinesie (4)

Q42 Weet u wat de oorzaak van de aanvallen bij uw hond is? Slechts een antwoord is mogelijk

- Idiopathisch (dit wil zeggen: we weten de oorzaak niet, vaak vermoedelijk genetisch). Maar er is geen oorzaak gevonden. (2)
- Metabool / toxisch / reactief (= de oorzaak ligt buiten de hersenen. Denk aan bijv een lever, nier of suikertekort). Er is dan bloedonderzoek uitgevoerd waaruit bleek dat de oorzaak dus niet idiopathisch is. (3)
- Structureel of secundair (= de oorzaak zit in de hersenen. Denk aan bijv trauma , ontsteking, tumor) (4)

Q43 Wie heeft deze diagnose gesteld?

- Ikzelf (1)
- Mijn dierenarts (2)
- De specialist (3)

Q45 Wie is de hoofdbehandelaar van de epilepsie bij uw hond?

- (Eerstelijns) dierenarts (1)
- Specialist veterinaire neurologie (2)
- Specialist veterinaire neurologie in opleiding (SIO) (3)
- Specialist interne geneeskunde (4)
- Niemand / ikzelf (5)

Einde blok: Eerste aanvallen

Start van blok: Aanvullende vragen

Q12 Aanvullende vragen over de aanvallen.

Q53 Hoe lang heeft het geduurd voordat u met een anti-epilepticum bent gestart: gerekend vanaf de eerste aanval die u heeft gezien.

- We zijn gelijk, de eerste week, na de eerste aanval begonnen met een medicatie. (1)
- We zijn (nog) niet begonnen met een medicatie. (2)
- We hebben gewacht: vul hier het aantal maanden tussen de eerste aanval en de start van de medicatie in. (3) __________________________________________________

Q54 Kunt u een trigger (aanleiding) aanwijzen voor het optreden van de aanvallen? Meerdere antwoorden zijn mogelijk.

- Stress (1)
- Sexuele opwinding (2)
- Weersinvloeden (3)
- Bezoek aan huis (4)
- Bezoek aan de dierenarts (5)
- Er is een duidelijk tijdstip van de dag aanwezig (6)
- Er is een duidelijke seizoensinvloed aanwezig (7)
- Ik herken geen trigger (10)
- Anders, ik vul het zelf in (11) __________________________________________________

| Pagina-einde |  |
| --- | --- |

| Pagina-einde |  |
| --- | --- |

Q58 Vaak zien we een inleidende fase. Dat is niet de aanval zelf maar een fase (seconden tot dagen voor de werkelijke aanval) voor de aanval. Herkent u deze bij uw hond?

- Ja (1)
- Nee (2)
- Weet ik niet (3)

Q59 Indien u een inleidende fase ziet: kunt u deze dan beschrijven?

- Misselijkheid (1)
- Braken (2)
- Speekselen / kwijlen (3)
- Rusteloosheid / hij is drukker (4)
- Hij/Zij zoekt contact met mij/ons (5)
- De hond wordt agressief (6)
- Hij doet anders, kijkt anders (7)

Deze vraag weergeven:

If Vaak zien we een inleidende fase. Dat is niet de aanval zelf maar een fase (seconden tot dagen vo... = Ja

Q60 Hoe lang voordat de aanval optreedt ziet u deze inleidende fase? Geef het svp weer in minuten.

________________________________________________________________

Q61 Kunt u het optreden van een aanval voorspellen?

- Ja, altijd (100% van de keren) (1)
- Ja, in 75% van de keren (2)
- Ja, in 50% van de keren (3)
- Ja, in 25% van de keren (4)
- Nee (5)

Q62 Heeft u ooit gezien dat uw hond zich bewust was van een aanval?

- Ja (1)
- Nee (2)

Q63 Wat doet uw hond veelal voordat een aanval optreedt?

- Hij/zij sliep (1)
- Hij/zij werd net wakker (2)
- Hij/zij was wakker maar lag in zijn mand/kleed te rusten (3)
- Liep buiten (4)
- Was aan het spelen (5)
- Was aan het sporten (6)
- Ik zie het nooit (7)

Q64 Kunt u contact met uw hond maken tijdens de aanval?

- Ja (1)
- Nee (2)
- Weet ik niet (3)

| Pagina-einde |  |
| --- | --- |

Q66 Hoe lang duren de aanvallen gemiddeld? Vul minuten in. Vul hier niet de tijd voor de aanval (inleidende fase) of na de aanval (post-ictale fase) in. Puur alleen de aanval.

________________________________________________________________

Q69	Hoe lang duurde de kortste aanval? Vul minuten in.

________________________________________________________________

Q68	Hoe lang duurde de langste aanval? Vul minuten in.

________________________________________________________________

Einde blok: Aanvullende vragen

Start van blok: Vragen over de aanvallen zelf

Q13 Vragen over de aanvallen zelf

Q70 Hieronder staan meerdere zaken genoemd die bij een aanval kunnen optreden. Wilt u dat wat u regelmatig ziet aanklikken? Meerdere antwoorden zijn mogelijk.

- Verkrampen van de poten en de nek (1)
- Neervallen (2)
- Tijdens de aanval ligt de hond op een zijde (3)
- Fiets bewegingen met de poten (4)
- Draaien van het hoofd (5)
- Kauwbewegingen met de bek (6)
- Trekkingen van de aangezichtsspieren (7)
- Urineverlies (8)
- Ontlastingsverlies (9)
- Speekselen (10)
- Verwijding van de pupillen (11)
- Rondjes lopen (12)
- Staartjagen (13)
- Bewustzijnsverlies (14)
- Staren (15)
- Contact zoeken met mensen (16)
- Tegen meubels / andere zaken oplopen (17)
- Ziet slecht / tijdelijk zichtverlies (18)
- Blaffen (19)
- Angst (20)
- Agressie (21)

Q71 Zien alle aanvallen er steeds hetzelfde uit?

- Ja (1)
- Nee (2)

Deze vraag weergeven:

If Zien alle aanvallen er steeds hetzelfde uit? = Nee

Q85 U gaf nee als antwoord. Kunt u aangeven waarom?

________________________________________________________________

Q72 Is uw hond tussen de aanvallen door normaal? Dus in de periodes dat er geen aanvallen zijn?

- Ja (1)
- Nee (2)
- Weet ik niet (3)
- Indien u het niet weet of nee als antwoord geeft kunt u dit dan verduidelijken? (4) __________________________________________________

Q73 Heeft u de indruk dat de linker of juist de rechterhelft van het lichaam tijdens een aanval meer actief is (denk bijvoorbeeld aan het steeds naar een zijde bewegen van het hoofd)?

- Ja er is een verschil (of het links of rechts is maakt voor dit antwoord niet uit) (1)
- Nee ik zie geen links-rechts verschil (2)
- Weet ik niet (3)

Q74 Kunt u de aanval verkorten?

- Ja (1)
- Nee (2)
- Weet ik niet (3)
- Indien ja, wat doet u dan? (4) __________________________________________________

Einde blok: Vragen over de aanvallen zelf

Start van blok: Post-ictale fase

Q14 Vragen over de fase na de aanval: de post-ictale fase. Deze fase kan seconden tot dagen duren.     **Vaak lopen ze dan doelloos rond, zijn afwezig, zien vaak slecht en zo verder,..**

Q75 De post-ictale fase is de fase na de aanval. Deze fase is soms niet herkenbaar, soms juist dagen aanwezig. Heeft uw hond een post-ictale fase?

- Ja (1)
- Nee (2)
- Weet ik niet (3)

Q76 Heeft u de indruk dat uw hond zich de aanval herinnert?

- Ja (1)
- Nee (2)
- Weet ik niet (3)

Q77 Hoe lang duurt het voordat de post-ictale fase voorbij is? Geef het weer in minuten.

________________________________________________________________

Q78 Reageert de hond op u tijdens de post-ictale fase?

- Ja (1)
- Nee (2)
- Weet ik niet (3)

Q79 Wat ziet u tijdens de post-ictale fase?

- De hond is moe (1)
- De hond loopt doelloos rond (2)
- De hond lijkt blind te zijn (3)
- De hond is agressief (4)
- De hond wil gelijk drinken (5)
- De hond wil gelijk eten (6)
- De hond wil wandelen (7)
- De hond wil niets doen en blijft liggen (8)
- De hond braakt (9)
- De hond gaat zich uitrekken (10)
- Overige (geen nadere toelichting) (11) __________________________________________________

Einde blok: Post-ictale fase

Start van blok: Dierenarts

Q15 Wat uw dierenarts zoal heeft gedaan?

Q80 Wat heeft uw dierenarts zoal gedaan om te komen tot de diagnose?

- Klinisch onderzoek (1)
- Hartecho (2)
- Bloedonderzoek (3)
- ECG (hartfilmpje) (4)
- ECHO hart (5)
- EEG (Electro-encephalogram - hersenonderzoek) (6)
- MRI scan van de hersenen (7)
- CT scan van de hersenen (8)
- Hersenvochtonderzoek: CSF / CSV onderzoek / liquor punctie (9)
- Overige (geef nadere toelichting) (10) __________________________________________________
- Urineonderzoek (11)

Q81 Heeft uw hond, naast de epilepsie, ook andere problemen?

- Ja (1)
- Nee (2)

Deze vraag weergeven:

If Heeft uw hond, naast de epilepsie, ook andere problemen? = Ja

Q79 Wat zijn de andere problemen van uw hond?

________________________________________________________________

Einde blok: Dierenarts

Start van blok: Aantallen, ernst, controle

Q83 Hoeveel aanvallen heeft uw hond de laatste maanden gehad? Tel alle aanvallen bij elkaar op. Ook de aanvallen die binnen een cluster optreden.

- De laatste 12 maanden heeft mijn hond ... aanvallen gehad (1) __________________________________________________
- De laatste 6 maanden heeft mijn hond ... aanvallen gehad (2) __________________________________________________
- De laatste 3 maanden heeft mijn hond ... aanvallen gehad (3) __________________________________________________
- De laatste maand heeft mijn hond ... aanvallen gehad (4) __________________________________________________

Q87 Heeft uw hond ook last van clusters? Onder een cluster verstaan we dat uw hond binnen een tijdsbestek van 24 uur meerdere aanvallen krijgt.

- Ja (1)
- Nee (2)

Deze vraag weergeven:

If Heeft uw hond ook last van clusters? Onder een cluster verstaan we dat uw hond binnen een tijdsbe... = Ja

Q88 Hoeveel clusteraanvallen* heeft uw hond de laatste maanden gehad? Vul hier alleen de clusters in.

- De laatste 12 maanden heeft mijn hond ... clusteraanvallen gehad (1) __________________________________________________
- De laatste 6 maanden heeft mijn hond ... clusteraanvallen gehad (2) __________________________________________________
- De laatste 3 maanden heeft mijn hond ... clusteraanvallen gehad (3) __________________________________________________
- De laatste maand heeft mijn hond ... clusteraanvallen gehad (4) __________________________________________________

Q84 Heeft uw hond ook last van status epilepsie? Onder een status verstaan we een aanval die langer duurt dan 10 minuten of een serie van aanvallen die zo kort op elkaar volgen dat de hond niet kan herstellen. Een status is een levensbedreigende situatie.

- Ja (1)
- Nee (2)

Deze vraag weergeven:

If Heeft uw hond ook last van status epilepsie? Onder een status verstaan we een aanval die langer d... = Ja

Q89 Hoe vaak is er in de laatste maanden sprake geweest van een status epilepticus?

- De laatste 12 maanden is er ... keer sprake geweest van een status epilepticus (1) __________________________________________________
- De laatste 6 maanden is er ... keer sprake geweest van een status epilepticus (2) __________________________________________________
- De laatste 3 maanden is er ... keer sprake geweest van een status epilepticus (3) __________________________________________________
- De laatste maand is er ... keer sprake geweest van een status epilepticus (4) __________________________________________________

| Pagina-einde |  |
| --- | --- |

Q90 De ernst van de tonisch clonische aanvallen beoordeel ik als: (met 0 niet ernstig en 10 zeer ernstig)	Sleep de schuifregelaar naar de gewenste positie

|  | 0 | 1 | 2 | 3 | 4 | 5 | 6 | 7 | 8 | 9 | 10 |
| --- | --- | --- | --- | --- | --- | --- | --- | --- | --- | --- | --- |

| 0=niet ernstig, 10=zeer ernstig (1) |  |
| --- | --- |

Q92 Indien aanwezig: De ernst van de focale aanvallen beoordeel ik als: (met 0 niet ernstig en 10 zeer ernstig)	Sleep de schuifregelaar naar de gewenste positie

|  | 0 | 1 | 2 | 3 | 4 | 5 | 6 | 7 | 8 | 9 | 10 |
| --- | --- | --- | --- | --- | --- | --- | --- | --- | --- | --- | --- |

| 0=niet ernstig, 10=zeer ernstig (1) |  |
| --- | --- |

| Pagina-einde |  |
| --- | --- |

Einde blok: Aantallen, ernst, controle

Start van blok: Medicatie

Q95 Welke medicatie, in tablet of capsule vorm,  gebruikt uw hond? (meerdere antwoorden mogelijk)

- Fenobarbital (merknamen: Phenoral, Phenoleptil en Soliphen) (1)
- Imepitoine (Pexion) (3)
- Kaliumbromide (merknamen: Epikal, Libromide) (4)
- Gabapentine (merknamen: Neurontin of Gabapentine) (7)
- Levetiracitam (merknamen: Keppra of levetiracetam) (8)
- Geen medicatie (9)

Q125 Welke extra medicatie (vaak nood) gebruikt uw hond? (meerdere antwoorden mogelijk)

- Neusspray midazolam (5)
- Diazepam, valium, stesolid (rectaal) (6)
- Levetiracitam (merknamen: Keppra of levetiracetam) (8)
- Gabapentine (merknamen: Neurontin of Gabapentine) (7)
- Geen medicatie (9)

Deze vraag weergeven:

If Welke medicatie, in tablet of capsule vorm,  gebruikt uw hond? (meerdere antwoorden mogelijk) != Geen medicatie

And En Welke medicatie, in tablet of capsule vorm,  gebruikt uw hond? (meerdere antwoorden mogelijk) q://QID95/SelectedChoicesCount is gelijk aan 1

Or Of Welke medicatie, in tablet of capsule vorm,  gebruikt uw hond? (meerdere antwoorden mogelijk) q://QID95/SelectedChoicesCount is groter dan 1

Q96 Welke neveneffecten van de medicatie ziet u bij uw hond? (er zijn meerdere antwoorden mogelijk)

- Sloomheid (1)
- Meer slapen (2)
- Rusteloosheid (3)
- Prikkelbaar (4)
- Wankel zijn / ongecoördineerd (5)
- Spierzwakte / zwakte (6)
- Toename eetlust (7)
- Gewichtstoename (8)
- Braken (9)
- Diarree (10)
- In huis poepen (11)
- Meer drinken (12)
- Meer plassen (13)
- Hoesten (14)
- Huiduitslag (15)
- GEEN. Mijn hond heeft geen bijwerkingen (16)

| Pagina-einde |  |
| --- | --- |

Q97 Maakt uw hond gebruik van alternatieve behandelingsmethoden?

- Ja (1)
- Nee (2)

Deze vraag weergeven:

If Maakt uw hond gebruik van alternatieve behandelingsmethoden? = Ja

Q98 Van welke alternatieve behandelingsmethode(n) maakt uw hond gebruik? (meerdere antwoorden mogelijk)

- Fytotherapie (1)
- CBD olie (2)
- CBD/THC olie (3)
- MCT olie (4)
- Speciaal dieet (5)
- Muziektherapie (6)
- Anders, namelijk... (7) __________________________________________________

Einde blok: Medicatie

Start van blok: Scores

Q99 Terugkijkend naar de periode voordat uw hond epilepsie kreeg, als die periode 100% goed was. Hoe scoort u uw hond dan nu? Het cijfer 0 drukt uit dat u de situatie erg slecht vindt en 100 betekent dat het erg goed gaat. 	Sleep de schuifregelaar naar de gewenste positie

|  | 0 | 10 | 20 | 30 | 40 | 50 | 60 | 70 | 80 | 90 | 100 |
| --- | --- | --- | --- | --- | --- | --- | --- | --- | --- | --- | --- |

| 0=het gaat erg slecht, 100=het gaat erg goed (1) |  |
| --- | --- |

Q102 De ernst van de aanvallen bij mijn hond is acceptabel voor mij. Sleep de schuifregelaar naar de gewenste positie. Met 0 het gaat erg goed en 10 het gaat erg slecht

|  | 0 | 1 | 2 | 3 | 4 | 5 | 6 | 7 | 8 | 9 | 10 |
| --- | --- | --- | --- | --- | --- | --- | --- | --- | --- | --- | --- |

| 0=het gaat erg goed, 10=het gaat erg slecht (1) |  |
| --- | --- |

Q103 Ik durf mijn hond alleen thuis te laten.	Sleep de schuifregelaar naar de gewenste positie. Met 0 geen enkel probleem en 10 ik durf hem/haar niet alleen te laten.

|  | 0 | 1 | 2 | 3 | 4 | 5 | 6 | 7 | 8 | 9 | 10 |
| --- | --- | --- | --- | --- | --- | --- | --- | --- | --- | --- | --- |

| 0=geen enkel probleem, 10=ik durf hem/haar niet alleen te laten (1) |  |
| --- | --- |

Q104 Het zorgen voor mijn hond met epilepsie beperkt mij in mijn dagelijkse bezigheden; het zorgt voor een afname van mijn eigen kwaliteit van leven.	Sleep de schuifregelaar naar de gewenste positie. Met 1 zegt u dat uw kwaliteit van leven op het dieptepunt zit. Met een 10 dat het geweldig gaat.

|  | 1 | 2 | 3 | 4 | 5 | 6 | 6 | 7 | 8 | 9 | 10 |
| --- | --- | --- | --- | --- | --- | --- | --- | --- | --- | --- | --- |

| 1=een waar dieptepunt, 10=het gaat echt heel goed (1) |  |
| --- | --- |

Q105 Het zorgen voor mijn hond met epilepsie is het waard. Sleep de schuifregelaar naar de gewenste positie. Met 0 zegt u dat het echt een probleem is en met 10 dat u het echt de moeite waard vindt.

|  | 0 | 1 | 2 | 3 | 4 | 5 | 6 | 7 | 8 | 9 | 10 |
| --- | --- | --- | --- | --- | --- | --- | --- | --- | --- | --- | --- |

| 0=een groot probleem, 10=geen enkel bezwaar (1) |  |
| --- | --- |

Q107 De neven-effecten van de medicatie bij mijn hond zijn acceptabel voor mij.	Sleep de schuifregelaar naar de gewenste positie. Met 0 geen probleem / geen bijwerkingen en 10 veel bijwerkingen: niet acceptabel.

|  | 0 | 1 | 2 | 3 | 4 | 5 | 6 | 7 | 8 | 9 | 10 |
| --- | --- | --- | --- | --- | --- | --- | --- | --- | --- | --- | --- |

| 0=geen probleem, 10=veel bijwerkingen: niet acceptabel (1) |  |
| --- | --- |

Q110 Scoor de kwaliteit van leven van uw hond. Sleep de schuifregelaar naar de gewenste positie. Met 1 zeer slecht en 10 uitstekend.

|  | 1 | 2 | 3 | 4 | 5 | 6 | 6 | 7 | 8 | 9 | 10 |
| --- | --- | --- | --- | --- | --- | --- | --- | --- | --- | --- | --- |

| 1=zeer slecht, 10=uitstekend (1) |  |
| --- | --- |

| Pagina-einde |  |
| --- | --- |

Q111 Welke fase van epilepsie vindt u de grootste aanslag op de kwaliteit van leven van uw hond?

- Inleidende fase / prodromale fase: Dit is een fase van abnormaal gedrag, mogelijk gepaard gaande met braken; het duurt enkele seconden tot dagen. (1)
- De aanval / ictus: Dit is een fase van bewustzijnsverlies en krampen; dit kan gepaard gaan met blaffen, kwijlen, plassen en poepen; dit duurt seconden tot minuten. (2)
- De herstelfase / post-ictale fase: Dit is een fase waarin de hond soms niet kan lopen, slaperig is, onrustig en mogelijk agressief is; het duurt seconden tot weken. (3)
- Geen specifieke fase (4)

Einde blok: Scores

Start van blok: Einde

Q113 Hartelijk bedankt voor uw medewerking aan dit onderzoek. Indien u interesse heeft in de resultaten van het onderzoek, dan kunt u hieronder uw e-mailadres doorgeven, dan zult u de uiteindelijke onderzoeksresultaten ontvangen. Dat kan meerdere maanden duren. Indien u vragen en/of opmerkingen heeft, kunt u die hier plaatsen.

________________________________________________________________

________________________________________________________________

________________________________________________________________

________________________________________________________________

________________________________________________________________

Einde blok: Einde
