# Supplementary material for: Phenotypic characterisation, quality of life and prevalence of idiopathic epilepsy and epilepsy of unknown cause in the nine Dutch dog breeds
Source: Front Vet Sci. 2026 Aug 27;13:1897489. doi: 10.3389/fvets.2026.1897489 (PMC13587619; doi:10.3389/fvets.2026.1897489)
Supplement: Supplementary file 2 [file Data_Sheet_2.docx]

Epilepsy research - English Survey (TRANSLATED)

Start van blok: intro

Q1
Dear owner,

INTRO, BLABLA

Completing the survey takes 30 minutes. That's a long time but we think the answers will really help us further. Before you continue, it is best to get your dog's pedigree, vaccination booklet, epilepsy diary and medication. Not every question requires an answer. Some do. All data is treated confidentially and is not shared with third parties. We therefore apply the rules regarding the AVG / GDPR. If you have any questions and/or comments, please post them on the last page of the survey. You may be unsure whether your dog has epilepsy. Then watch these videos beforehand. Both focal and generalized seizures are possible. If you first want to read more about this, you can go to this website for more information: www.veterinair-neuroloog.nl
 
Examples of focal seizures: https://youtu.be/AZhL9-_MvEM
 
An example of a generalised tonic-clonic seizure: https://youtu.be/6ticKGNgojw
 
Next to this we rarely see paroxsymal dyskinesia. An example is visible here:  https://youtu.be/96UMxPZH1OA
 
It could be that you dog has both. If there are questions contact us

Many thanks for your help.
 
dr. Paul Mandigers (Veterinaire Neurologie, Universiteit Utrecht & Evidensia Dierenziekenhuis Arnhem)

Q114 Do you permit us to use the information you are going to provide?

- Yes I do (1)
- No I do not (2)

Deze vraag weergeven:

If Do you permit us to use the information you are going to provide? = No I do not

Q115 You have not agreed to the use of your entered data, hence you have reached the end of this survey.

Ga naar: Einde enquête Als You have not agreed to the use of your entered data, hence you have reached the end of this survey. Weergegeven

Einde blok: intro

Start van blok: Informatie eigenaar

Q8 Information about yourself. Entry is not mandatory but we think an important factor is the interaction between dog and owner. Hence these questions.

Q5 Which city and country do you live? (you are not obliged to answer this question)

________________________________________________________________

Q6 What is your email address? (Input is not mandatory, but it is desirable so that we can reach you afterwards in case of ambiguities).

________________________________________________________________

Q116 How would you describe your own life situation. Several questions follow. Please go through them all. I am,...

- Single (1)
- Together with my partner (2)
- Family with children (3)

Q117 Where do you live?

- I live in a rural / village and it is quiet here (1)
- I live in a rural / village but it can be busy (2)
- It is quite busy here but it is not a city (3)
- I live in a city but it is quiet here (4)
- I live in a city and it is rather busy here (5)
- Different, I will give my own text (6) __________________________________________________

Q122 Your home situation

- It is always quiet at home (1)
- There is always something to do but it isn't busy at home (2)
- It is always busy at home (3)
- It is always vey busy at home and lots of things happen (4)
- Different, I will give my own textl (5) __________________________________________________

Q118 I regard myself to be

- A very calm balanced person (1)
- A very calm balanced person but I am busy (2)
- A very busy but still balanced person (3)
- An extremely busy and sometimes hurried person (4)
- A rather stressfull life (5)
- Extremely stressfull life (6)
- Different, I will give my own text (7) __________________________________________________

Q120 If you live with others (a partner or with children). If not applicable, skip this question.

- My house/roommates are calm and balanced people (1)
- My house/roommates are busy but balanced persons (2)
- My house/roommates are very busy but still balanced persons (3)
- My roommates are extremely busy and sometimes very rushed (4)
- My roommates make it a rather stressful existence (5)
- My roommates make it extremely stressful (6)
- Diffenet, I will give my own text (7) __________________________________________________

Q121 If you have several pets: which is applicable. If not applicable, skip this question.

- I have one or more dogs (1)
- I have one or more cats (2)
- I have one or more cats and dogs (3)
- I do not have any other pets (4)
- Different, I will give my own text (8) __________________________________________________

Q123 If you have several pets: which is applicable. If not applicable, skip this question.

- There is no hassle between my various pets (1)
- There is rarely a bit of hassle between my various pets (2)
- There is always a lot of hassle between my various pets (3)
- I do not have any other pets (4)
- Different, I will give my own text (8) __________________________________________________

Einde blok: Informatie eigenaar

Start van blok: Vragen hond, vaccinatie etc

Q9 Questions about your dog, vaccination history etc.

Q17 What is your dog's name?

________________________________________________________________

Q18 Does your dog have a pedigree and from which breed?

Q19 If applicable: what is your dog's pedigree name?

________________________________________________________________

Q20 Pedigree number

________________________________________________________________

Q21 Chipnumber of your dog?

________________________________________________________________

Q22 What is your dog's date of birth? You may write it down or fill it in like this: dd/mm/yyyy

________________________________________________________________

________________________________________________________________

________________________________________________________________

________________________________________________________________

________________________________________________________________

Q23 What is the gender of your dog?

- Male (1)
- Female (2)

Q24 Has your dog been spayed or neutered?

- Yes (1)
- No (2)

Q25 If spayed or castrated: do you remember the approximate date on which the spaying/neutering took place? (dd/mm/yyyy)

________________________________________________________________

________________________________________________________________

________________________________________________________________

________________________________________________________________

________________________________________________________________

| Pagina-einde |  |
| --- | --- |

Q27 Was your dog vaccinated before he/she had the first epileptic seizure?

- Yes (1)
- No (2)
- If the answer is YES. Which brand? You can find this in the vaccination booklet. (3) __________________________________________________
- If the answer is YES. When was that (4) __________________________________________________

Q28 Did your dog receive deworming tablets before he/she had the first epileptic seizure?

- Yes (1)
- No (2)
- If YES: do you also know what the brand of this deworming was? (3) __________________________________________________
- If YES: do you also know when that was approximately? (4) __________________________________________________

Q29 Did you treat your dog with anti-flea/tick products before he/she had the first epileptic seizure?

- Yes (1)
- No (2)
- If yes which brand was it? (3) __________________________________________________
- Of yes, when was this? (4) __________________________________________________

Q30 What was your dog's diet before having the first epileptic seizure (Indicate brand if possible)?

- Type of food 1 (1) __________________________________________________
- Type of food 2 (2) __________________________________________________
- Type of food 3 (3) __________________________________________________

Q31 Has your dog's diet changed?

- Yes (3)
- No (4)
- If yes, which brand? (5) __________________________________________________

Q32 Before having the first epileptic seizure, did your dog have a serious illness? If in doubt, always fill in Yes and state which disease it was.

- Yes (2)
- No (3)
- If yes what had happened? (4) __________________________________________________

Einde blok: Vragen hond, vaccinatie etc

Start van blok: Woonsituatie

Q10 General questions about your dog

Q35 How would you describe your dog's character? (multiple answers are correct)

- Vivid (1)
- Cheerfull (2)
- Calm (3)
- Nervous (4)
- Anxious (5)
- Aggressive (6)
- Other, namely (7) __________________________________________________

Q36 Do you keep your dog outside or mainly inside?

- Inside (1)
- Outside (2)
- A combination of both (3)
- Other, namely (4)

Q37 How many hours per day is your dog supervised by you or one of your housemates?

- Less than 5 hours a day (1)
- Five to 10 hours a day (2)
- Ten to 15 hours a day (3)
- 15 to 20 hours a day (4)
- More than 20 hours a day (5)

Einde blok: Woonsituatie

Start van blok: Eerste aanvallen

Q11 Questions concerning the first seizures

Q126 When did you see the first seizure in your dog? If you do not remember the exact date, please give an approximate date. (dd/mm/yyyy)

________________________________________________________________

________________________________________________________________

________________________________________________________________

________________________________________________________________

________________________________________________________________

Q127 When did the most recent seizure occur?

________________________________________________________________

________________________________________________________________

________________________________________________________________

________________________________________________________________

________________________________________________________________

Q40 Did you dog suffer from more than one seizure?

- Yes (1)
- No (2)

Q41 How many seizures has your dog had to date? Enter a number.

________________________________________________________________

Q44

Dogs can have several types of epileptic seizures.

Both focal and generalized seizures are possible. If you first want to read more about this, you can go to this website for more information: https://www.veterinair-neurologisch.nl/gezondheids/epilepsy

Examples of focal seizures can be seen here: https://youtu.be/AZhL9-_MvEM
 
An example of a tonic-clonic seizure: https://youtu.be/6ticKGNgojw

 Next to this we see rarely paroxsymal dyskinesia: https://youtu.be/96UMxPZH1OA
 
It could be that you see combinations within your dog. If there are questions do not hesitate to contact us.

Q124 What kind of seizures does your dog have?

- Generalized tonic-clonic seizures. These are attacks in which the dog usually lies on its side, has cramps and cycling movements, twitches with the mouth, as a rule you do not have contact with him / her and saliva, urine and faeces may occur. (1)
- Focal seizures. These are, for example, seizures where only part of the body shows the abnormality. Examples are: pulling with the lips, salivating, just a paw, etc. (2)
- A combination of both tonic-clonic seizures and the focal seizures. (3)
- Other, namely,.... (4) __________________________________________________

Q42 Do you know what causes your dog's seizures? Only one answer is possible

- Idiopathic (i.e. we don't know the cause, often presumably genetic). But no cause has been found. (2)
- Metabolic / toxic / reactive (= the cause lies outside the brain. Think of a liver, kidney or sugar deficiency, for example). A blood test was then carried out which showed that the cause is not idiopathic. (3)
- Structural or secondary (= the cause is in the brain. Think of trauma, inflammation, tumor, for example) (4)
- Differently, namely,....... (5) __________________________________________________

Q43 Who made this diagnosis?

- I did (1)
- My own veterinarian (2)
- The sepcialist (3)
- Differently, namely,.... (4) __________________________________________________

Q45 Who is the veterinarian who treats you dog?

- Firstline practitioner (1)
- Specialist in Veterinary Neurology (2)
- Resident in Veterinary Neurology (3)
- Specialist Internal Medicine (4)
- Other, namely,... (5) __________________________________________________

Deze vraag weergeven:

If Who is the veterinarian who treats you dog? = Specialist in Veterinary Neurology

Or Who is the veterinarian who treats you dog? = Resident in Veterinary Neurology

Or Who is the veterinarian who treats you dog? = Specialist Internal Medicine

Or Of Who is the veterinarian who treats you dog? Text Response Bevat specialist

Q46 How did you end up with the specialist in Veterinary Neurology?

- I arranged it myself (1)
- My local veterinarian advised me (2)
- Other, namely (3) __________________________________________________

Einde blok: Eerste aanvallen

Start van blok: Aanvullende vragen

Q12 Additional questions about the seizures

Q47 Is your dog still alive?

- Yes (1)
- No (2)

Deze vraag weergeven:

If Is your dog still alive? = No

Q48 At what age (in years) did your dog died?

________________________________________________________________

Deze vraag weergeven:

If Is your dog still alive? = No

Q49 What was the cause fo death?

- It was related to the epilepsy (1)
- Other, namely,.... (3) __________________________________________________

| Pagina-einde |  |
| --- | --- |

Q50 How many seizures did you observe the FIRST year that your dog started having the seizures

- Number of seizures a year (1) __________________________________________________
- Number of seizures during the first 6 months (2) __________________________________________________
- Number of seizures during the first 3 months (3) __________________________________________________
- Number of seizures during the first month (4) __________________________________________________
- Number of seizures during the first week (5) __________________________________________________

Q128 How many seizures did you observe the LAST year?

- Not applicale, this is the first year (6) __________________________________________________
- Number of seizures a year (1) __________________________________________________
- Number of seizures during the first 6 months (2) __________________________________________________
- Number of seizures during the first 3 months (3) __________________________________________________
- Number of seizures during the first month (4) __________________________________________________
- Number of seizures during the first week (5) __________________________________________________

Q52 How do you rate the severity of the attacks NOW compared to the first attacks. You can now use the slider to indicate whether it has become less or worse. If you put the bar in the middle it has remained the same. Less to the left and more to the right. Zero means not serious, Ten means very serious.

|  | 0 | 1 | 2 | 3 | 4 | 5 | 6 | 7 | 8 | 9 | 10 |
| --- | --- | --- | --- | --- | --- | --- | --- | --- | --- | --- | --- |

| 0=not serious,5=equal, 10=very serious (1) |  |
| --- | --- |

Q53 How long did it take you to start taking an anti-epileptic: counted from the first seizure you saw.

- We are right, the first week, after the first attack started with a medication. (1)
- We haven't started any medication yet. (2)
- We waited: enter the number of months between the first attack and the start of the medication. (3) __________________________________________________

Q54 Can you pinpoint a trigger for the seizures to occur? Multiple answers are correct.

- Stress (1)
- Sexual arisement (2)
- The weather (3)
- Visits at homes (4)
- Visit to the vet (5)
- It is always the same moment (7)
- I do not recognize a trigger (10)
- Other, I will fill it in myself (11) __________________________________________________

| Pagina-einde |  |
| --- | --- |

Q55 You indicated that your dog has been spayed or neutered. Was this done AFTER the dog developed seizures?

- Yes after that the siezures were first seen (1)
- No prior (2)
- I do not remember anymore (3)

Deze vraag weergeven:

If You indicated that your dog has been spayed or neutered. Was this done AFTER the dog developed se... = Yes after that the siezures were first seen

Q56 Has the seizure frequency changed after neutering?

- Yes it dropped in number (1)
- No it did not (2)
- It actually increased (3)
- Other, namely,.... (4) __________________________________________________

Q57 Is your dog related to other dogs with epilepsy?

- Yes (1)
- No (2)
- I do not know (3)
- If yes, what is the relationship? (4) __________________________________________________

| Pagina-einde |  |
| --- | --- |

Q58 Often we see an introductory phase. That is not the attack itself, but a phase (seconds to days before the actual attack) before the attack. Do you recognize this in your dog?

- Yes (1)
- No (2)
- I do not know (3)

Q59 If you see an introductory phase, can you describe it?

- Nausea (1)
- Vomiting (2)
- Drooling (3)
- Restlessness (4)
- He/She makes contact with us (5)
- The dog becomes aggressive (6)
- Other, namely,.... (7) __________________________________________________

Deze vraag weergeven:

If Often we see an introductory phase. That is not the attack itself, but a phase (seconds to days b... = Yes

Q60 How long before the attack occurs do you see this introductory phase? Please state it in minutes.

________________________________________________________________

Q61 Can you predict the occurrence of a seizure?

- Yes, always (100% of the time) (1)
- Yes roughly in 75% of the time (2)
- Yes roughly in 50% of the time (3)
- Yes roughly in 25% of the time (4)
- No (5)

Q62 Have you ever seen your dog aware of a seizure?

- Yes (1)
- No (2)
- Other, namely.... (3) __________________________________________________

Q63 What does your dog usually do before a seizure occurs?

- Asleep (1)
- Just woke up (2)
- He/she was awake but still on his/her basket (3)
- Waling outside (4)
- Was playing (5)
- Was busy (6)
- I never see it (7)
- Other, namely,... (8) __________________________________________________

Q64 Can you make contact with your dog during the attack?

- Yes (1)
- No (2)
- I do not know (3)

Deze vraag weergeven:

If Can you make contact with your dog during the attack? = Yes

Q65 If you make contact during an attack. How does the dog react?

- Awake, responds normal (1)
- Different but seems to react (2)
- Unresponsive (3)
- Other, namely,... (4) __________________________________________________

| Pagina-einde |  |
| --- | --- |

Q66 How long do the attacks last on average? Enter minutes. Do not enter the time before the attack (preliminary phase) or after the attack (post-ictal phase). Purely attack only.

________________________________________________________________

Q69 How long did the shortest attack last? Enter minutes.

________________________________________________________________

Q68 How long did the longest attack last? Enter minutes.

________________________________________________________________

Einde blok: Aanvullende vragen

Start van blok: Vragen over de aanvallen zelf

Q13 Questions related to the seizures

Q70 Below are several things that can occur during an attack. Do you want what you see click on regularly? Multiple answers are correct.

- Cramping with his legs and head (1)
- Drops down (2)
- During a seizure the dog lies on his side (3)
- Cycle movements with the legs (4)
- Turns with his head (5)
- Chewing movements with his jaws (6)
- Twitching of the facial muscles (7)
- Urinary loss (8)
- Defeacation loss (9)
- Drooling (10)
- Dilation of the pupils (11)
- Circling (12)
- Tail chasing (13)
- Loss of consciousness (14)
- Gazing / Stare (15)
- Seeks attention (16)
- Walk onto the furniture (17)
- Seems to see less / appears to be blind (18)
- Barking (19)
- Anxiety (20)
- Agression (21)
- Other, namely,...) (22) __________________________________________________

Q71 Do all seizures always look the same?

- Yes (4)
- No (2)
- I no, can you indicate why? (3) __________________________________________________

Q72 Is your dog normal between seizures? So in the periods when there are no seizures?

- Yes it is otherwise a normal dog (1)
- No (2)
- I do not know (3)
- If you don't know or answer no, can you clarify? (4) __________________________________________________

Q73 Do you have the impression that the left or the right half of the body is more active during an attack (think, for example, of the head always moving to one side)?

- Yes (1)
- No I do not see a difference (2)
- I do not know (3)

Q74 Can you shorten a seizure?

- Yes (1)
- No (2)
- I do not know (3)
- If yes what do you do? (4) __________________________________________________

Einde blok: Vragen over de aanvallen zelf

Start van blok: Post-ictale fase

Q14
Questions about the post-seizure phase: the post-ictal phase. This phase can last seconds to days.

They often walk around aimlessly, are absent, often see poorly and so on, ..

Q75 Does your dog have a post-ictal phase?

- Yes (1)
- No (2)
- I do not know (3)

Q76 Do you have the impression that your dog remembers the attack?

- Yes (1)
- No (2)
- I do not know (3)

Q77 How long does it take for the post-ictal phase to pass? Display it in minutes.

________________________________________________________________

Q78 Does the dog respond to you during the post-ictal phase?

- Yes (1)
- No (2)
- I do not know (3)

Q79 What do you observe during this post-ictal phase?

- The dog is tired (1)
- The dogs is walking restlessness around (2)
- The dog appears to see poorly (3)
- The dog is aggressive (4)
- The dog wants to drink (5)
- The dogs wants to eat (6)
- The dog wants to walk (7)
- The dogs doesn't want to do anything and lies still (8)
- The dog vomits (9)
- The dog starts stretching (10)
- Other, namely,.. (11) __________________________________________________

Einde blok: Post-ictale fase

Start van blok: Dierenarts

Q15 What did your veterinarian do?

Q80 What did your vet do to arrive at the diagnosis?

- A physical exam (1)
- Ultrasound heart (2)
- Bloodexamination (3)
- ECG (Electrocardiogram) (4)
- EEG (Electro-encephalogram) (6)
- MRI scan of the brain (7)
- CT scan of the brain (8)
- CSF examination / liquor punctie (9)
- Other, namely,...... (10)

Q81 Does your dog have another illness?

- Yes (1)
- No (2)
- If yes, please indicate what (3) __________________________________________________

Einde blok: Dierenarts

Start van blok: Geslacht specifiek

Q16 Specific questions concerning bitches and sires

Deze vraag weergeven:

If What is the gender of your dog? = Female

Q82 At what age did your bitch come into heat for the first time?

- She hasn't come into heat yet (1)
- Not applicable (2)
- We saw it the first time: (3) __________________________________________________

Deze vraag weergeven:

If What is the gender of your dog? = Female

Q83 Is her heat regular?

- Yes (1)
- No (2)
- I do not know (3)
- Not applicable (4)

Deze vraag weergeven:

If What is the gender of your dog? = Female

Q84 Did you dog ever had pups?

- Yes (1)
- No (2)
- I do not know (3)
- If yes how often? (4) __________________________________________________

Deze vraag weergeven:

If What is the gender of your dog? = Male

Q85 For sires: does you dog have a normal sexual behaviour?

- Yes (1)
- No (2)
- I do not know (3)
- If not what is going on? (4) __________________________________________________

Deze vraag weergeven:

If What is the gender of your dog? = Male

Q86 For sires: do your dog have offspring?

- Yes (1)
- No (2)
- I do not know (3)
- If yes how many litters? (4) __________________________________________________

Einde blok: Geslacht specifiek

Start van blok: Aantallen, ernst, controle

Q87 How many seizures has your dog had in the last few months? Count the clusters* as one seizure. *A cluster is defined as seizures that follow one another (within 24 hours) where the dog has normal consciousness between seizures.

- For the last 12 months my dog has had ..... seizures (1) __________________________________________________
- For the last 6 months my dog has had ...... seizures (2) __________________________________________________
- For the last 3 months my dog has had ...... seizures (3) __________________________________________________
- During the last month my dog has had ...... seizures (4) __________________________________________________

Q88 Hoeveel clusteraanvallen* heeft uw hond de laatste maanden gehad?Vul hier alleen de clusters in. Indien u ze niet ziet vult u hier dus niets in.	* Met clusteraanvallen wordt bedoeld: aanvallen die op elkaar volgen (binnen 24 uur) maar waarbij de hond tussen de aanvallen door een normaal bewustzijn heeft.

- For the last 12 months my dog has had ...... clusters (1) __________________________________________________
- For the last 6 months my dog has had ...... clusters (2) __________________________________________________
- For the last 3 months my dog has had ...... clusters (3) __________________________________________________
- During the last month my dog has had ...... clusters (4) __________________________________________________

Q89 How often have you had a status epilepticus* in the last few months? * Status epilepticus means that the seizures follow each other within a very short time, with no recovery phase

- There has been ...... status epilepticus time in the last 12 months (1) __________________________________________________
- There has been ...... status epilepticus time in the last 6 months (2) __________________________________________________
- There has been ...... status epilepticus time in the last 3 months (3) __________________________________________________
- There has been ...... status epilepticus time in the last month (4) __________________________________________________

| Pagina-einde |  |
| --- | --- |

Q90 I rate the severity of the tonic-clonic seizures as: (with 0 not severe and 10 very severe) Drag the slider to the desired position

|  | 0 | 1 | 2 | 3 | 4 | 5 | 6 | 7 | 8 | 9 | 10 |
| --- | --- | --- | --- | --- | --- | --- | --- | --- | --- | --- | --- |

| 0=not severe, 10=very severe (1) |  |
| --- | --- |

Q92 If present: I rate the severity of the focal seizures as: (with 0 not severe and 10 very severe) Drag the slider to the desired position

|  | 0 | 1 | 2 | 3 | 4 | 5 | 6 | 7 | 8 | 9 | 10 |
| --- | --- | --- | --- | --- | --- | --- | --- | --- | --- | --- | --- |

| 0=not severe, 10=very severe (1) |  |
| --- | --- |

Q91 I can predict from the behavior of the dog when an attack is coming; my dog shows a changed behavior some time before the seizure. (with 0 disagree and 10 strongly agree) Drag the slider to the desired position

|  | 0 | 1 | 2 | 3 | 4 | 5 | 6 | 7 | 8 | 9 | 10 |
| --- | --- | --- | --- | --- | --- | --- | --- | --- | --- | --- | --- |

| 0=disagree, 10=strongly agree (1) |  |
| --- | --- |

| Pagina-einde |  |
| --- | --- |

Q93 How often does your dog come for a check-up with a vet or specialist?

- Once a week (1)
- every 2 weeks (2)
- Every 3 weeks (3)
- Once a month (4)
- Every two months (5)
- Every three months (6)
- Every four months (7)
- Every 5 months (8)
- Every 6 months (9)
- Once a year (10)
- Never (11)
- Other, namely,... (12)

Q94
In the past few months, how often did you have to go to a vet in the evenings or at weekends? related to your dog's epilepsy?

- The past 12 months,.... times (1) __________________________________________________
- The past 6 months,.... times (2) __________________________________________________
- The past 3 months,.... times (3) __________________________________________________
- The past month,.... times (4) __________________________________________________

Einde blok: Aantallen, ernst, controle

Start van blok: Medicatie

Q95 What medication, in tablet or capsule form, does your dog use? (multiple answers possible)

- Fenobarbital (brand names a.o.: Phenoral, Phenoleptil en Soliphen) (1)
- Imepitoine (Pexion) (3)
- Potassiumbromid (brand names: Epikal, Libromide) (4)
- Gabapentin (brand names: Neurontin of Gabapentine) (7)
- Levetiracitam (merknamen: Keppra of levetiracetam) (8)
- No medication (9)
- Other, namely,... (10) __________________________________________________

Q125 What additional (often emergency) medication does your dog take? (multiple answers possible)

- Nasalspray midazolam (5)
- Diazepam, valium, stesolid (rectal) (6)
- Levetiracitam (brand names: Keppra of levetiracetam) (8)
- Gabapentin (brand names: Neurontin of Gabapentine) (7)
- No medication (9)
- Other, namely,... (10) __________________________________________________

Deze vraag weergeven:

If What medication, in tablet or capsule form, does your dog use? (multiple answers possible) != No medication

And En What medication, in tablet or capsule form, does your dog use? (multiple answers possible) q://QID95/SelectedChoicesCount is gelijk aan 1

Or Of What medication, in tablet or capsule form, does your dog use? (multiple answers possible) q://QID95/SelectedChoicesCount is groter dan 1

Q96 What side effects of the medication do you see in your dog? (multiple answers are possible)

- Lethargy (1)
- More sleeping (2)
- Restlessness (3)
- Irritable (4)
- Incoordination (5)
- Muscle weakness (6)
- Increased appetite (7)
- Weight gain (8)
- Vomiting (9)
- Diarrhea (10)
- Defecating in house (11)
- Increased drinking (12)
- More ofte urinating (13)
- Coughing (14)
- Skinratch (15)
- None, my dog doesn't show any sidefeffects (16)
- Other, namely,... (17) __________________________________________________

| Pagina-einde |  |
| --- | --- |

Q97 Do you make use of an alternative treatment?

- Yes (1)
- No (2)

Deze vraag weergeven:

If Do you make use of an alternative treatment? = Yes

Q98 What alternative treatment method(s) does your dog use? (multiple answers possible)

- Phytotherapy (1)
- CBD oil (2)
- CBD/THC oil (3)
- MCT oil (4)
- Special diet (5)
- Music therapy (6)
- Other namely,. (7) __________________________________________________

Einde blok: Medicatie

Start van blok: Scores

Q99 Looking back to the period before your dog developed epilepsy, if that period was 100% correct. How do you rate your dog now? The number 0 indicates that you think the situation is very bad and 100 means that things are going very well. Drag the slider to the desired position

|  | 0 | 10 | 20 | 30 | 40 | 50 | 60 | 70 | 80 | 90 | 100 |
| --- | --- | --- | --- | --- | --- | --- | --- | --- | --- | --- | --- |

| 0=very bad, 100=all is perfect (1) |  |
| --- | --- |

Q101 In the last 3 months I have been concerned about the frequency of my dog's seizures. Drag the slider to the desired position. With 0 no worries and 10 very worried.

|  | 0 | 1 | 2 | 3 | 4 | 5 | 6 | 7 | 8 | 9 | 10 |
| --- | --- | --- | --- | --- | --- | --- | --- | --- | --- | --- | --- |

| 0=no worries, 10=lots of worries (1) |  |
| --- | --- |

Q102 The severity of my dog's seizures is acceptable to me. Drag the slider to the desired position. With 0 it is going very well and 10 it is going very badly

|  | 0 | 1 | 2 | 3 | 4 | 5 | 6 | 7 | 8 | 9 | 10 |
| --- | --- | --- | --- | --- | --- | --- | --- | --- | --- | --- | --- |

| 0=it goes quite well, 10=it goes poorly (1) |  |
| --- | --- |

Q103 I dare to leave my dog at home alone. Drag the slider to the desired position. With 0 no problem at all and 10 I don't dare to alt him/her alone.

|  | 0 | 1 | 2 | 3 | 4 | 5 | 6 | 7 | 8 | 9 | 10 |
| --- | --- | --- | --- | --- | --- | --- | --- | --- | --- | --- | --- |

| 0=no problem at all, 10=it is really a big problem (1) |  |
| --- | --- |

Q104 Taking care of my dog with epilepsy limits my daily activities; it causes a decrease in my own quality of life. Drag the slider to the desired position. With 1 you say that your quality of life is at its lowest point. With a 10 that it's going great.

|  | 1 | 2 | 3 | 4 | 5 | 6 | 6 | 7 | 8 | 9 | 10 |
| --- | --- | --- | --- | --- | --- | --- | --- | --- | --- | --- | --- |

| 1=terrible, 10=it is really going great (1) |  |
| --- | --- |

Q105 Taking care of my dog with epilepsy is worth it. Drag the slider to the desired position. With 0 you say it is really a problem and with 10 that you think it is really worth it.

|  | 0 | 1 | 2 | 3 | 4 | 5 | 6 | 7 | 8 | 9 | 10 |
| --- | --- | --- | --- | --- | --- | --- | --- | --- | --- | --- | --- |

| 0=a big issue, 10=really worth the effort (1) |  |
| --- | --- |

Q106 The administration of the medication to my dog causes problems. Drag the slider to the desired position. With 0 you say it's not a problem and with 10 it's really not a problem.

|  | 0 | 1 | 2 | 3 | 4 | 5 | 6 | 7 | 8 | 9 | 10 |
| --- | --- | --- | --- | --- | --- | --- | --- | --- | --- | --- | --- |

| 0=no problem, 10=really not a problem (1) |  |
| --- | --- |

Q107 The side effects of the medication on my dog are acceptable to me. Drag the slider to the desired position. With 0 no problem / no side effects and 10 many side effects: not acceptable.

|  | 0 | 1 | 2 | 3 | 4 | 5 | 6 | 7 | 8 | 9 | 10 |
| --- | --- | --- | --- | --- | --- | --- | --- | --- | --- | --- | --- |

| 0=not a problem, 10=many side-effects, not acceptable (1) |  |
| --- | --- |

Q108 The cost of epilepsy treatment is acceptable to me. Drag the slider to the desired position. With 0 not acceptable/really a problem and 10 very acceptable

|  | 0 | 1 | 2 | 3 | 4 | 5 | 6 | 7 | 8 | 9 | 10 |
| --- | --- | --- | --- | --- | --- | --- | --- | --- | --- | --- | --- |

| 0=not acceptable, 10=very acceptable (1) |  |
| --- | --- |

Q109 Is it a problem for you to go for a consultation with the vet or specialist (for example, for check-ups, research of the blood concentrations of the medication)? Drag the slider to the desired position. With 0 no problem at all and with 10 you say it is a big problem.

|  | 0 | 1 | 2 | 3 | 4 | 5 | 6 | 7 | 8 | 9 | 10 |
| --- | --- | --- | --- | --- | --- | --- | --- | --- | --- | --- | --- |

| 0=no problem, 10=a very big problem (1) |  |
| --- | --- |

Q110 Score your dog's quality of life. Drag the slider to the desired position. With 1 very bad and 10 excellent.

|  | 1 | 2 | 3 | 4 | 5 | 6 | 6 | 7 | 8 | 9 | 10 |
| --- | --- | --- | --- | --- | --- | --- | --- | --- | --- | --- | --- |

| 1=very poor, 10=excellent (1) |  |
| --- | --- |

| Pagina-einde |  |
| --- | --- |

Q111 Which stage of epilepsy do you consider to be the biggest drain on your dog's quality of life?

- Introductory phase / prodromal phase: This is a phase of abnormal behavior, possibly accompanied by vomiting; it takes a few seconds to days. (1)
- The seizure / ictus: This is a phase of loss of consciousness and cramps; this may be accompanied by barking, drooling, urinating and defecating; this takes seconds to minutes. (2)
- The recovery phase / postictal phase: This is a phase where the dog is sometimes unable to walk, sleepy, restless and possibly aggressive; it takes seconds to weeks. (3)
- No specific phase (4)

Q112 Unfortunately, it is very difficult to obtain complete freedom of attack. But what do you consider reasonably acceptable?

- One aweek (1)
- One every two weeks (2)
- One every three weeks (3)
- One every month (4)
- One every two months (5)
- One every three months (6)
- One every four months (7)
- One every 5 months (8)
- One every 6 months (9)
- One a year (10)
- No seizures at all (11)

Einde blok: Scores

Start van blok: Einde

Q113 Thank you very much for your participation in this research. If you are interested in the results of the survey, please enter your email address below and you will receive the final survey results. If you have any questions and/or comments, please post them here.

________________________________________________________________

________________________________________________________________

________________________________________________________________

________________________________________________________________

________________________________________________________________

Einde blok: Einde
